# A Paradoxical Tumor-Suppressor Role for the Rac1 Exchange Factor Vav1 in T Cell Acute Lymphoblastic Leukemia

**DOI:** 10.1016/j.ccell.2017.10.004

**Published:** 2017-11-13

**Authors:** Javier Robles-Valero, L. Francisco Lorenzo-Martín, Mauricio Menacho-Márquez, Isabel Fernández-Pisonero, Antonio Abad, Mireia Camós, María L. Toribio, Lluis Espinosa, Anna Bigas, Xosé R. Bustelo

**Affiliations:** 1 Centro de Investigación del Cáncer, CSIC - University of Salamanca, 37007 Salamanca, Spain; 2 Instituto de Biología Molecular y Celular del Cáncer, CSIC - University of Salamanca, 37007 Salamanca, Spain; 3 Centro de Investigación Biomédica en Red de Cáncer (CIBERONC), CSIC - University of Salamanca, 37007 Salamanca, Spain; 4 Hospital Sant Joan de Déu, 08950 Esplugues de Llobregat, Spain; 5 Centro de Biología Molecular Severo Ochoa, CSIC - Madrid Autonomous University, 28049 Madrid, Spain; 6 Institut Hospital del Mar d’Investigacions Mèdiques, 08003 Barcelona, Spain

**Keywords:** Notch1, Rho GTPases, Cbl-b, TLX, lymphoma, animal models, gene expression profiling

## Abstract

Rho guanine exchange factors (GEFs), the enzymes that stimulate Rho GTPases, are deemed as potential therapeutic targets owing to their protumorigenic functions. However, the understanding of the spectrum of their pathobiological roles in tumors is still very limited. We report here that the GEF Vav1 unexpectedly possesses tumor-suppressor functions in immature T cells. This function entails the noncatalytic nucleation of complexes between the ubiquitin ligase Cbl-b and the intracellular domain of Notch1 (ICN1) that favors ICN1 ubiquitinylation and degradation. Ablation of Vav1 promotes ICN1 signaling and the development of T cell acute lymphoblastic leukemia (T-ALL). The downregulation of Vav1 is essential for the pathogenesis of human T-ALL of the TLX^+^ clinical subtype, further underscoring the suppressor role of this pathway.

## Highlights

• *Vav1*-deficient mice are highly prone to early cortical, Notch1-dependent T-ALL
• Vav1 acts as a tumor suppressor by controlling active Notch1 fragment (ICN1) levels
• This is a noncatalytic function of Vav1 mediated by the Cbl-b E3 ubiquitin ligase
• *VAV1* silencing is important for human TLX^+^ T-ALL pathogenesis

## Graphical Abstract

**Figure:**
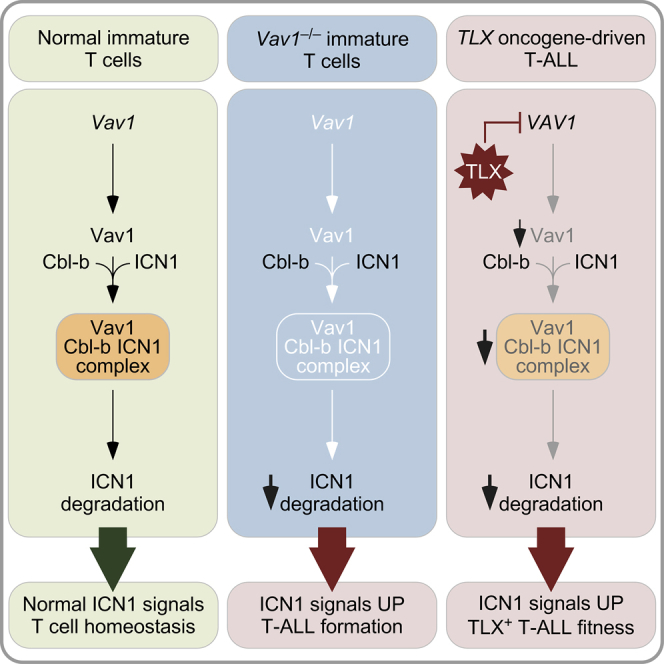

## Teaser

Robles-Valero et al. find that Vav1 facilitates binding of Cbl-b to the intracellular domain of Notch1 (ICN1) and promotes ICN1 degradation. Loss of Vav1 induces T cell acute lymphoblastic leukemia (T-ALL) by increasing ICN1 signaling, and TLX inhibits Vav1 expression to stimulate ICN1 signaling in TLX^+^ T-ALL.

## Significance

**Rho GEFs, including Vav1, are traditionally regarded as protumorigenic elements in tumors. Contrary to this paradigm, we demonstrate here that Vav1 can perform tumor-suppressor functions in immature T cells. This catalysis-independent activity depends on the formation of Vav1-Cbl-b-ICN1 complexes that facilitate the Cbl-b-mediated degradation of ICN1. This pathway is active in many T-ALL cell lines regardless of the mutational status of Notch1. We also show that the repression of this tumor-suppressor pathway by transcriptional factors of the TLX family is required for the fitness of human TLX**^**+**^ **T-ALL. These data challenge the concept of the monodimensional implication of Rho GEFs in protumorigenic pathways, unveil a function for Vav1, and provide insights into human TLX**^**+**^ **T-ALL pathogenesis.**

## Introduction

The human genome encodes ≈70 Rho guanine exchange factors (GEFs) involved in the catalytic stimulation of Rho guanosine triphosphatases (GTPases). This drug-targetable activity, together with the key roles played by Rho GTPases in cancer-related processes, has led to the consideration of these enzymes as potential drug targets. Reinforcing this view, studies using cell lines and animal models have clearly established direct connections between Rho GEF activity and protumorigenic events (Vigil et al., 2010). Despite this evidence, the structural and functional complexity of these proteins indicates that the univocal relationship between Rho GEF activity and protumorigenic functions might not always occur. In this context, some features of these pathways suggest that, at least theoretically, some Rho GEF subsets could antagonize cell transformation. Thus, the discovery of loss-of-function *RHOA* gene mutations in human tumors suggests the possibility that RhoA-specific GEF subsets could exert suppressor roles in cells that have not yet acquired those mutations (Zandvakili et al., 2017). The same concept applies to GEFs that stimulate RhoB, a GTPase with tumor-suppressing activities (Vigil et al., 2010, Zandvakili et al., 2017). Given their multidomain structure, it is also possible that GEFs could promote tumor-suppression pathways via GTPase-independent mechanisms.

Vav1 is a hematopoietic-specific GEF that epitomizes the structural and functional complexity of the Rho GEF family. Thus, it harbors calponin-homology (CH), acidic (Ac), catalytic Dbl-homology (DH), pleckstrin-homology (PH), zinc-finger (ZF), SH2, and SH3 domains that have regulatory (CH, Ac, SH2, SH3), catalytic (DH, PH, ZF regions), and adaptor (CH, SH3) functions. As a result, Vav1 can engage catalysis-dependent and -independent pathways during cell signaling (Bustelo, 2014). Extensive genetic evidence using both cell lines and knockout mice support the implication of Vav1 in cell transformation. In fact, its discovery was possible due to the transforming activity displayed by an oncogenic mutant version in focus formation assays (Bustelo, 2014). Its connection with protumorigenic events has been further reinforced by the recent discovery of potential *VAV1* gain-of-function mutations in adult T cell leukemia and lung tumors (Abate et al., 2017, Boddicker et al., 2016, Campbell et al., 2016, Kataoka et al., 2015). However, contrary to this canonical view, it has been observed that the loss of Vav1 favors the progressive emergence of T cell tumors in aging mice (Ruiz et al., 2009). The cause of this unexpected phenotype remains unknown.

The Notch1 pathway is frequently involved in human T cell acute lymphoblastic leukemia (T-ALL). The ADAM and γ-secretase proteases cleave this receptor in a ligand-dependent manner under physiological conditions, leading to the release of its cytoplasmic ICN1 tail. ICN1 then translocates to the nucleus, interacts with RBPJκ, and stimulates expression of cell fate-, metabolic-, and proliferation-related genes. This transcriptional program is eventually shut down by ICN1 degradation, a step regulated by the E3 ubiquitin ligase Fbxw7. This tight regulation is frequently lost owing to gain- and loss-of-function mutations in *NOTCH1* or *FBXW7* genes in T-ALL, respectively (Van Vlierberghe and Ferrando, 2012). However, these mutations seem to require additional genetic lesions to drive T-ALL, including gain-of-function alterations in transcriptional factors such as LYL1, HOXA, TAL1, TLX1, and TLX3 (Van Vlierberghe and Ferrando, 2012).

We have recently found that carcinogen-exposed young *Vav1*^−/−^ mice develop quite aggressive early cortical T-ALL with very short latency periods. The investigation of this rather paradoxical effect led us to discover a Vav1-dependent tumor-suppressor pathway involved in ICN1 regulation in immature T cells.

## Results

### The *Vav1* Gene Deficiency Promotes Immature T Cell Tumors in Mice

**Figure 1:**
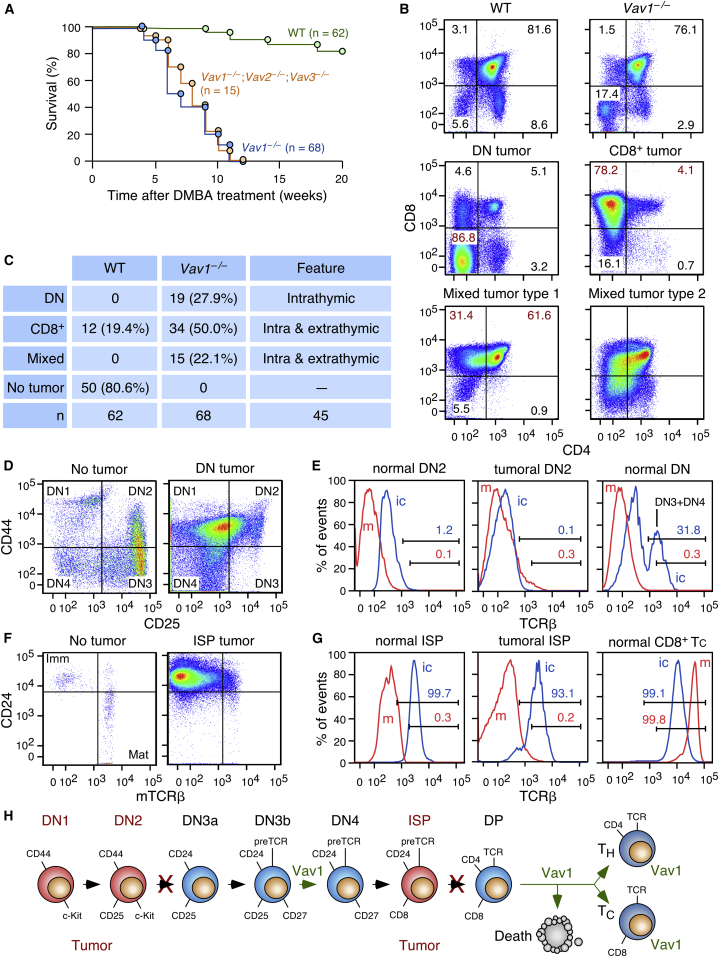
*Vav1* Deficiency Promotes Immature T Cell Tumors in Mice (A) Survival rates of mice of indicated genotypes upon DMBA administration. (B) Surface immunophenotype of thymocytes from control and *Vav1*^−/−^ mice (top two panels) and from representative cases of the main pathological classes found in tumor-bearing *Vav1*^−/−^ mice (other panels). Numbers in each quadrant indicate the relative percentage of each cell population. (C) Summary of tumor types obtained at the time of the death of mice. (D) Flow cytometry of DN-gated thymocytes from a healthy (not treated with DMBA) and a DN tumor-bearing (DMBA-treated) *Vav1*^−/−^ mouse upon staining with antibodies to CD44 and CD25. Similar data were obtained in 15 independent determinations. (E) Flow cytometry showing percentages of cells staining positive for intracellular TCRβ (ic, blue) and membrane TCRβ (m, red) in the DN2 (two left panels) and the total DN population (right panel) from a healthy (not treated with DMBA) and a DN tumor-bearing (DMBA-treated) *Vav1*^−/−^ mouse. Similar data were obtained in 15 independent determinations of DN tumor-bearing mice. (F) Flow cytometry of CD8-gated thymocytes from a healthy (not treated with DMBA) and a CD8^+^ tumor-bearing (DMBA-treated) *Vav1*^−/−^ mouse upon staining with antibodies to CD24 and TCRβ. Similar data were obtained in 40 independent determinations of CD8^+^ tumor-bearing mice. Imm, immature ISP cells; Mat, mature cytotoxic T cells. (G) Flow cytometry showing percentage of immature single positive (ISP) and CD8^+^ cytotoxic T (Tc) cells staining positive for icTCRβ (blue) and mTCRβ (red) in indicated cell populations of a healthy (not treated with DMBA) and a CD8^+^ tumor-bearing (DMBA-treated) *Vav1*^−/−^ mouse. Similar data were obtained in 40 independent determinations of CD8^+^ tumor-bearing mice. (H) Scheme of T cell differentiation showing stages (red) and developmental blocks (crosses) detected in tumors from *Vav1*^−/−^ mice. The steps dependent on canonical Vav1 functions are in green. DP, double-positive (CD4^+^CD8^+^) cells; T_H_, CD4^+^ helper T cells. See also Figures S1 and S2.

While addressing the role of Vav proteins in tumorigenic processes, we found that *Vav1*^−/−^ mice become sick quite rapidly upon the administration of carcinogens such as 7,12-dimethylbenz[*a*]anthracene (DMBA), N-nitroso-N-methylurea, and urethane (Figure 1A, data not shown). As a result, *Vav1*-deficient mice become terminally ill 10–12 weeks after exposure to the carcinogen (Figure 1A). Necropsies revealed the presence of highly enlarged thymi lacking the typical bilobular morphology (Figure S1A). These thymi also displayed effaced corticomedullary boundaries (Figure S1B), an increase in thymus weight (Figure S1C), and a “starry night” histology characterized by the presence of macrophages containing large numbers of engulfed dead cells (Figure S1B). This phenotype is exclusively driven by *Vav1* deficiency since compound *Vav1*^−/−^;*Vav2*^−/−^;*Vav3*^−/−^ animals have survival curves similar to their *Vav1*^−/−^ counterparts (Figure 1A).

The most frequent tumors detected in *Vav1*^−/−^ mice are composed of either CD4^–^CD8^–^ (double negative; DN) or CD8^+^ T cells (Figures 1B, 1C, and S1D). Tumors containing a mixture of those two immunophenotypes (mixed tumor type 1 and type 2) are detected at lower frequencies (Figures 1B and 1C). DN tumor cells display an intermediate immunophenotype between the DN1 (CD44^+^CD25^–^) and the DN2 (CD44^+^CD25^+^) differentiation stage (Figure 1D). Consistent with this, they lack intracellular (ic) and membrane expression (m) of the T cell receptor (TCR) β subunit (Figure 1E). CD8^+^ tumor cells are CD24^+^ (Figure 1F), icTCRβ^+^, and mTCRβ^–^ (Figure 1G), thus indicating that they derive from the abnormal expansion of immature single CD8-positive (ISP) cells (Figure 1H). The DN and CD8^+^ tumor populations differ in their ability to disseminate outside the thymus, since we could only find peripheral cancer cells at high frequency (45% of cases, n = 45) in CD8^+^ tumor-bearing animals (Figures 1C, S1E, and S1F). Similar immunophenotype distributions were observed in tumors from N-nitroso-N-methylurea- and urethane-treated *Vav1*^−/−^ mice (data not shown).

The DMBA-induced tumors develop very quickly in *Vav1*^−/−^ mice, as assessed by the detection of abnormally expanded DN (Figure S2A) and CD8^+^ (Figure S2B) populations as early as 1 week after ending the DMBA treatments in ≈35% of all the animals surveyed (Figure S2C). This phenotype reached a penetrance of 70% and 100% 4 and 12 weeks later, respectively (Figure S2C). The effect of the growth of these tumor cells in the overall size of the thymus (Figures S2D and S2E) and number of thymocytes (Figure S2F) is also apparent 4 weeks after finishing the carcinogenic treatment. DMBA-exposed WT mice develop T cell tumors with longer latencies and lower frequencies (Figure 1A). These tumors are CD8^+^CD24^+^icTCRβ^+^ cells and harbor a significant pool (≈30%) of mTCRβ^+^ cells (Figures 1C and S2G). Taken together, these results indicate that *Vav1* acts as a tumor-suppressor gene at the DN1-DN2 and ISP T cell developmental stages (Figure 1H). It is unlikely that this is a reflection of a canonical function, since the known Vav1 GEF and adaptor activities are associated with thymocyte selection events taking place at the DN and CD4^+^CD8^+^ differentiation stages and, later on, with the antigenic responses of mature T cells (Figure 1H).

### *Vav1*^−/−^ Tumors Show Notch1-like Functional Signatures

**Figure 2:**
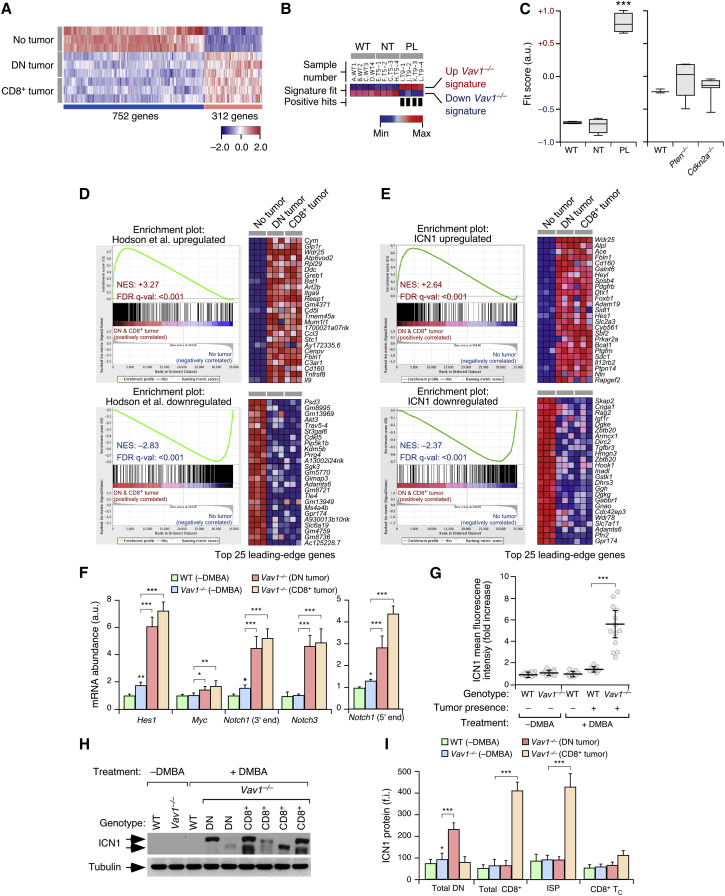
*Vav1*^−/−^ Tumors Show Notch1-like Functional Features (A) Transcripts commonly upregulated (red) and downregulated (blue) in the DN and CD8^+^ tumors arising in DMBA-treated *Vav1*^−/−^ mice. As comparative control, we used thymocytes from untreated *Vav1*^−/−^ mice (No tumor). Rows represent independent replicas. Total number of transcripts is indicated at the bottom. (B) Heatmap of upregulated and downregulated “common” *Vav1*^−/−^ tumor gene signatures enrichment scores calculated using ssGSEA for transcriptomes of thymocytes from WT mice or from nontumorigenic (NT) and preleukemic (PL) *Zfp36l1*^−/−^;*Zfp36l2*^−/−^ mice. Samples with a high signature fit are indicated by vertical black bars. Enrichment scores are depicted on a dark blue (lowest) to dark red (highest) scale. (C) Box plot of the “common” *Vav1*^−/−^ tumor gene signature fit score in indicated experimental groups. Boxes represent the central 50% of the data (from the lower 25th percentile to the upper 75th percentile), lines inside boxes represent the median (50th percentile), and whiskers are extended to the most extreme data point that is no more than 1.5 times the interquartile range from the box edge. ^∗∗∗^p ≤ 0.001 (according to Tukey's honest significance difference [HSD] test). (D and E) GSEA for the “common” *Vav1*^−/−^ tumor gene expression matrix (D and E; left graphs) using as gene sets the differentially expressed genes from *Zfp36l1*^*−/−*^; *Zfp36l2*^*−/−*^ mice (D) and ICN1-transformed CD4^+^CD8^+^TCRα/β^+^ cells (E). The expression profile of the top 25 leading-edge genes in the upregulated (D and E; right top clusters) and downregulated (D and E; right bottom clusters) gene sets in the transcriptome of thymocytes from healthy (No tumor), DN tumor-bearing (DN tumor), and CD8^+^ tumor-bearing (CD8^+^ tumor) *Vav1*^−/−^ mice is shown. The normalized enrichment scores (NES) and false discovery rate values (FDR, using q values) are indicated inside each GSEA graph. q-val, q value. (F) Abundance of indicated transcripts (bottom) in unfractionated thymic cells from control and tumor-bearing mice (segregated according to the immunophenotype of tumor cells). Values are given relative to the expression of each transcript in samples obtained from WT controls (n = 15 animals per class analyzed). (G) Flow-cytometry determination of ICN1 abundance in samples from indicated mouse cohorts. Each point represents the measurement of an individual mouse (n = 13 [WT − DMBA], 13 [*Vav1*^−/−^ − DMBA], 9 [WT + DMBA, no tumor], 7 [WT + DMBA, tumor positive], and 13 [*Vav1*^−/−^ + DMBA, tumor positive] animals). (H) Western blot (WB) showing abundance of ICN1 (top) and tubulin α (loading control, bottom) in total thymic extracts from indicated mice and experimental conditions. (I) Flow-cytometry determination of ICN1 abundance in indicated cell populations (bottom) and animal cohorts (inset). f.i., mean fluorescence intensity relative to the isotype-matched control antibody. In (F), (G), and (I), data represent the mean ± SEM. Statistical values obtained using either the Student’s t test (F and I) or Mann-Whitney test (G) are given relative to untreated WT controls or indicated experimental pairs (in brackets). ^∗^p ≤ 0.05, ^∗∗^p ≤ 0.01, ^∗∗∗^p ≤ 0.001. See also Figure S3; Tables S1 and S2.

*Vav1*^−/−^ tumor cells exhibit large transcriptional changes that involve the deregulation of “common” (Figure 2A and Table S1), DN-specific (Table S1), and CD8^+^-specific (Table S1) gene sets. We focused the subsequent analyses on the “common” gene signature class, since we surmised that it could give clues about the transformation process of both the *Vav1*^−/−^ DN and CD8^+^ tumor subpopulations. Standard functional annotation methods did not yield any obvious functional hint (Table S1). However, when compared with microarray datasets from other mouse tumors using single-sample gene set enrichment analyses (ssGSEA) and GSEA, we found that the “common” gene set shows high similarity to the transcriptome previously described for preleukemic thymocyte populations from *Zfp36l1*^−/−^;*Zfp36l2*^−/−^ mice (Figures 2B–2D). Zfp36L proteins antagonize T cell transformation through the binding to and inhibition of translation of *Notch1* transcripts (Hodson et al., 2010). This fact suggested that the loss of Vav1 could be associated with the spurious upregulation of the Notch1 pathway. Buttressing this hypothesis, the “common” *Vav1*^−/−^ tumor gene signature is also similar to the transcriptome of T-ALL cells generated upon ectopic expression of ICN1 in mouse bone marrow precursors (Figure 2E). This resemblance is not due to the activation of programs common to most cancer cells because the *Vav1*^−/−^ tumor gene signature does not overlap with the transcriptome of tumor T cells obtained from mice defective in tumor suppressors such as Pten and Cdkn2a (Figure 2C).

In agreement with these data, we detected high amounts of transcripts commonly upregulated in Notch1-driven T-ALL such as *Hes1*, *Myc*, *Notch1*, and *Notch3* in *Vav1*^−/−^ DN and CD8^+^ tumor cells using qRT-PCR (Figure 2F). The increase in *Notch1* mRNA abundance is seen using primers for both the 5′ and 3′ end of its cDNA (Figure 2F), indicating enhanced transcription from the WT locus rather than spurious expression of an ICN1-encoding mRNA found in some T-ALL (Jeannet et al., 2010). The activation of the Notch1 pathway goes in parallel with exacerbated amounts of ICN1 in the tumor cells (Figures 2G and 2H). Flow cytometry demonstrated the presence of high ICN1 levels in the DN and ISP cells that originate the tumors (Figure 2I). Sequencing of genomic DNA fragments from 20 independent tumors indicated that the upregulation of ICN1 is not linked to the emergence of *Notch1*, *Fbxw7*, or *Pten* mutations commonly found in T-ALL (Table S2).

No statistically significant changes in the amount of ICN1 (Figure 2G) and ICN1 downstream gene targets (Figure S3A) are detected in tumor T cells from DMBA-treated WT mice, indicating that the deregulation of this cascade is intrinsic to the *Vav1* deficiency. By contrast, we did find a small increase in the abundance of both ICN1 (Figure 2I) and some of its specific downstream transcripts (*Hes1*, *Notch1*; Figure 2F) in both DN populations and unfractionated thymocytes from untreated *Vav1*^−/−^ mice, respectively. This upregulation is too weak to be detected in total thymic extracts by immunoblotting (Figure 2H). These results indicate that the Notch1 pathway becomes deregulated in *Vav1*^−/−^ DN cells and that the tumorigenic process further accentuates this pathogenic event. This is a very early event because exacerbated ICN1 signaling is already observed in *Vav1*^−/−^ mice in the first week after the DMBA treatment (Figures S3B–S3D).

### *Vav1*^−/−^ T-ALL Is Notch1 Dependent

**Figure 3:**
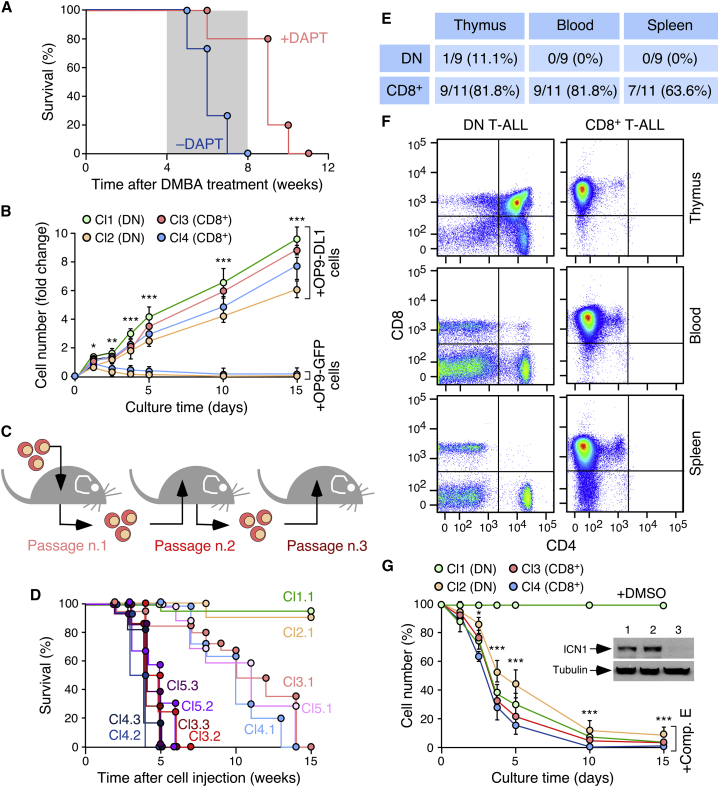
*Vav1*^−/−^ T-ALL Is Notch1 Dependent (A) Survival curves of DMBA-treated *Vav1*^*−/−*^ mice with (n = 5) and without (n = 4) DAPT administration (treatment period shaded in gray). (B) Proliferation of indicated tumor cell clones (Cl1–Cl4, see inset for color code) from *Vav1*^−/−^ mice in the presence of either OP9-GFP or OP9-DL1 cell layers. The immunophenotype of each clone is indicated in the inset. Similar results were obtained with 3 and 6 independent clones of the DN and CD8^+^ immunophenotype, respectively. (C) Scheme of the experiments performed in (D). (D) Survival curves of WT mice upon serial injections of indicated *Vav1*^−/−^ T-ALL clones (n = 5 per clone). Clones 1–4 are those shown in (B). Clone 5 (Cl5) is an additional CD8^+^ tumor cell clone. The number (x) and cycle of injection (y) of each clone are indicated using the notation Clx.y. (E) Number and percentage of *Vav1*^−/−^ DN and CD8^+^ T-ALL clones that recreated the T-ALL in passage 1 according to flow-cytometry analyses carried out in indicated tissues of terminally ill recipient animals. (F) Detection of T-ALL in mice injected with DN (n = 9) and CD8^+^ (n = 11) tumor cells in the indicated tissues based on CD4 and CD8 expression. (G) Survival of *Vav1*^−/−^ T-ALL cell clones cultured on OP9-DL1 cells either in the absence (+DMSO) or presence of Compound E (+Comp. E). Inset shows the abundance of ICN1 in one of these clones (Cl3) upon isolation from mice (1), culturing on OP9-DL1 cells (2), and after 5 days of Compound E treatment (3). In (B) and (G), data represent mean ± SEM. ^∗^p ≤ 0.05, ^∗∗^p ≤ 0.01, ^∗∗∗^p ≤ 0.001 relative to time-0 controls (Student's t test).

The implication of the Notch1 pathway in tumors arising in *Vav1*^−/−^ mice was confirmed by several lines of evidence. Firstly, we showed that the death of DMBA-treated *Vav1*^−/−^ mice can be delayed by administration of the γ-secretase inhibitor DAPT (Figure 3A). Secondly, we demonstrated that the long-term culture of *Vav1*^−/−^ DN and CD8^+^ tumor cells requires the presence of feeder layers of mouse bone marrow stromal cells expressing the Notch1 ligand Delta1 (Figure 3B). DN and CD8^+^ tumor cells preserve the original immunophenotype in these cultures (not shown) and, in the case of CD8^+^ cells, can regenerate a highly disseminated T-ALL condition when transplanted into recipient WT mice (Figures 3C–3F). These tumorigenic properties are further increased upon serial cycles of isolation, culturing, and reinjection into mice (Figures 3C and 3D). However, the cells maintain dependency on the Delta1-expressing feeder cells for propagation in culture (not shown). Consistent with these observations, we found that the viability of *Vav1*^−/−^ DN and CD8^+^ tumor cells is impaired by the addition of a γ-secretase inhibitor (Compound E) to the cultures (Figure 3G). This response is associated with the elimination of ICN1 in those cells (Figure 3G, inset). These results indicate that carcinogen-treated *Vav1*^*−/−*^ mice develop Notch1-driven DN and CD8^+^ TCR^–^ T-ALL.

### Vav1 Regulates ICN1 Degradation

**Figure 4:**
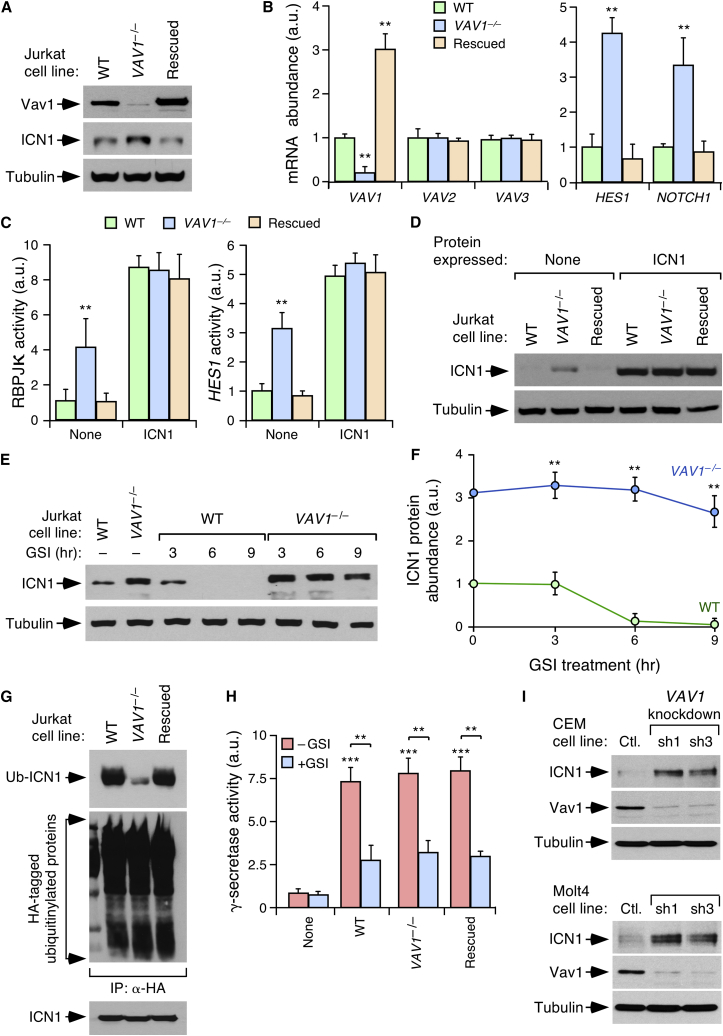
Vav1 Regulates ICN1 Degradation (A) Abundance of Vav1, ICN1, and tubulin α in total cellular lysates (TCL) from indicated cells. Rescued, a stable pool of *VAV1*^−/−^ Jurkat cells in which Vav1^WT^ was re-expressed. (B) Abundance of selected mRNAs in indicated Jurkat cells (n = 3). (C) Activity of RBPJκ-responsive (left) and *HES1* (right) promoters in indicated cells. Values are given relative to WT cells (n = 3). (D) Abundance of endogenous and ectopic ICN1 in TCLs from cells used in (C) (upper panel). Endogenous tubulin α was used as loading control (bottom panel). (E) Abundance of ICN1 (top) and tubulin α (bottom) in TCLs from indicated cells and conditions (n = 3). (F) Quantification of ICN1 abundance according to the data gathered in (E) (n = 3). (G) Cellular extracts from Jurkat cells coexpressing HA-ubiquitin and ICN1 were immunoprecipitated (IP) with antibodies to HA to determine the amount of ubiquitinylation of ectopic ICN1 (top) and endogenous proteins (middle) by immunoblot. Equal amounts of ICN1 expression in cells were confirmed by WB analysis using TCLs (bottom) (n = 3). Top and bottom panels were blotted with antibodies to ICN1. Middle panel was blotted with antibodies to the HA epitope. Ub, ubiquitinylated. (H) Presenilin activity in indicated Jurkat cells (bottom) and assay conditions (inset) (n = 3). (I) Abundance of endogenous ICN1 in TCLs from CEM (top panels) and Molt4 (bottom panels) cells expressing a control (Ctl.) or two independent (sh1 and sh3) *VAV1* shRNAs (n = 3). In (B), (C), (F), and (H), data represent mean ± SEM. ^∗∗^p ≤ 0.01, ^∗∗∗^p ≤ 0.001 using Student's t test (B, C, and F) and Mann-Whitney test (H). See also Figure S4.

We took advantage of Jurkat cells, a T-ALL cell line widely used in the characterization of the catalytic and adaptor functions of Vav1 in lymphocytes, to start dissecting the signaling connection between the Vav1 and Notch1 signaling pathways. To find out whether the observations made in mice could be recapitulated in this system, we first compared the status of the endogenous Notch1 route in the parental cell line and a *VAV1*^−/−^ derivative generated by homologous recombination techniques. As control, we used a *VAV1*^−/−^ Jurkat cell line ectopically expressing wild-type Vav1 (Vav1^WT^). We confirmed the expected Vav1 expression status in each of these cell lines by immunoblotting (Figure 4A; for statistics see Figure S4A) and qRT-PCR (Figure 4B). By contrast, the amount of the *VAV2* and *VAV3* mRNA does not change in any of these cells (Figure 4B). Similarly to *Vav1*^−/−^ T-ALL (Figures 2 and S3), we found that *VAV1*^−/−^ Jurkat cells exhibit increased amounts of ICN1 protein (Figures 4A and S4A) as well as of *HES1* and *NOTCH1* transcripts (Figure 4B) when compared with controls. They also show chronic stimulation of the endogenous ICN1-RBPJκ transcriptional complex, as assessed by gene reporter assays using vectors in which the expression of the luciferase gene was under the direct regulation of either RBPJκ binding sites or the *HES1* promoter (Figures 4C and 4D). These molecular alterations correlate with the detection of longer half-lives (Figures 4E and 4F) and defective ubiquitinylation (Figures 4G and S4A) of ICN1 in Vav1-deficient Jurkat cells. By contrast, we could not detect any significant effect of Vav1 deficiency on the activity of the presenilin complex that promotes the final proteolytic cleavage of Notch1 (Figure 4H). All the foregoing alterations are eliminated when Vav1^WT^ is expressed in *VAV1*^−/−^ cells (Figures 4A–4C and S4A), indicating that they are directly caused by the Vav1 deficiency. In agreement with this, we found that the short hairpin RNA (shRNA)-mediated elimination of the endogenous *VAV1* mRNA also results in the upregulation of ICN1 signaling in Jurkat cells (Figure S4). Interestingly, these experiments indicated that ICN1 upregulation does not require the complete depletion of Vav1 in cells.

Jurkat cells show a number of alterations in the Notch1 route, including an activating mutation in the Notch1 juxtamembrane domain and a hemizygous loss-of-function mutation (R505C) in Fbxw7 (https://humantallcelllines.wordpress.com). This suggests that Vav1 can affect the stability of ICN1 even in cells that have lost the normal regulation of this pathway. Confirming this idea, we found that the knockdown of *VAV1* also leads to the upregulation of ICN1 levels in both CEM (carrying heterozygous mutations in Fbxw7 [R465H] and the Notch1 heterodimerization domain [HD]) and Molt4 (bearing heterozygous mutations targeting both the HD and the proline-glutamic-serine-threonine [PEST] motif of Notch1) T-ALL cells (Figures 4I and S4A).

### Vav1 Modulates ICN1 in a Noncatalytic, Cbl-b-Dependent Manner

**Figure 5:**
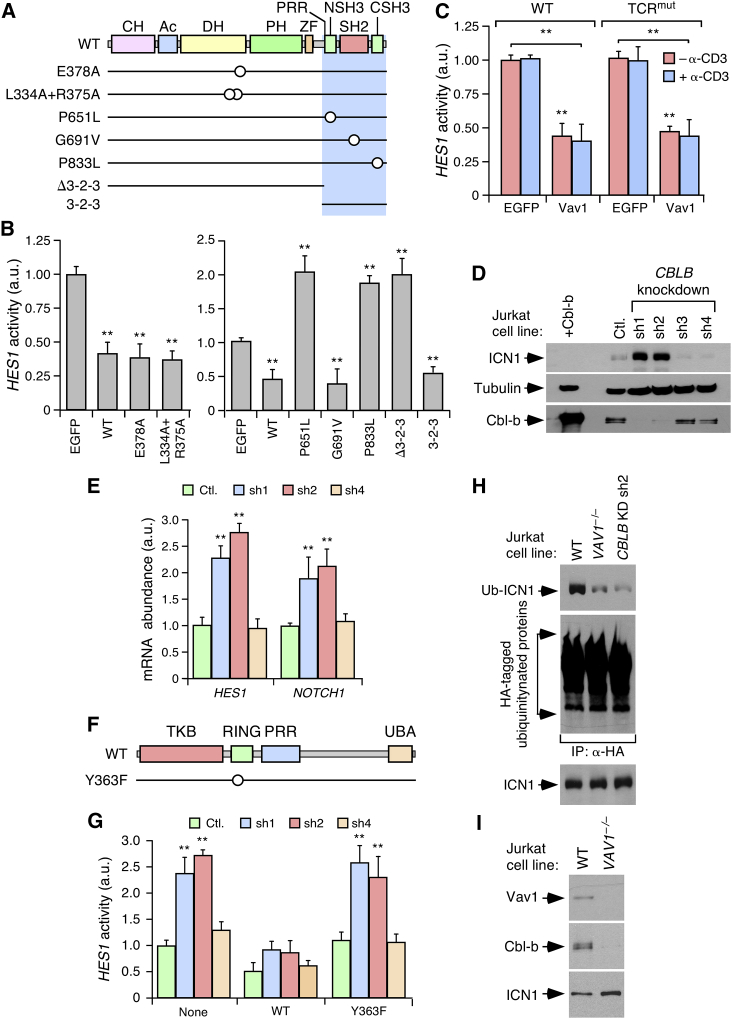
Vav1 Modulates ICN1 in a Cbl-b-Dependent Manner (A) Vav1 mutants used (point mutations depicted as open circles). The minimal region for the Vav1-dependent regulation of the Notch1 route is shaded in blue. (B) *HES1* promoter activity of *VAV1*^−/−^ Jurkat cells expressing the indicated EGFPs (bottom) (n = 5). (C) *HES1* promoter activity in nonstimulated (−α-CD3) and stimulated (+α-CD3) WT and TCR^mut^ Jurkat cells expressing the indicated EGFPs (bottom) (n = 3). (D) Abundance of ICN1, tubulin α, and Cbl-b in Jurkat cells expressing a control (Ctl.) and four independent (sh1 to sh4) *CBLB* shRNAs. Determinations were done by WB using either TCLs (ICN1, tubulin α) or immunoprecipitated Cbl-b. +Cbl-b, WT Jurkat cells ectopically expressing Cbl-b^WT^ (n = 3). (E) Abundance of indicated transcripts in cells used in these experiments (n = 3). Cells are designated as in (D). (F) Structure of Cbl-b and localization of the Y363F mutation. TKB, tyrosine kinase binding domain; RING, RING domain; UBA, ubiquitin-associated region. (G) *HES1* promoter activity in indicated cells upon transfection with an empty vector (None) or plasmids expressing indicated Cbl-b proteins (n = 3). Cells are designated as in (D). (H) ICN1 ubiquitinylation in indicated cells following the approach described in Figure 4G (n = 3). KD, knockdown. (I) Detection of endogenous Vav1 (top panel) and Cbl-b (middle panel) in immunoprecipitates of ICN1 (bottom panel) in indicated Jurkat cells. In (B), (C), (E), and (G), data represent mean ± SEM. ^∗∗^p ≤ 0.01 (Student’s t test). See also Figure S5.

We next expressed a variety of mouse EGFP-tagged Vav1 mutant proteins (Figure 5A) to identify the Vav1 structural domains involved in ICN1 regulation. Using the ability of these proteins to rescue normal *HES1* promoter activity when expressed in *VAV1*^−/−^ Jurkat cells as a readout, we found that Vav1 proteins with inactivating mutations in the catalytic (E378A, L334A + R375A) and SH2 (G691V) domains exhibit rescuing activities similar to Vav1^WT^ (Figures 5B and S5A). By contrast, this rescue activity is abolished when using Vav1 proteins lacking the entire SH3-SH2-SH3 region or bearing inactivating mutations in either the most N-terminal (NSH3, P651L) or C-terminal (CSH3, P833L) SH3 domains (Figures 5B and S5A). In fact, the expression of these mutant proteins results in the elevation of *HES1* promoter activity in *VAV1*^−/−^ cells (Figure 5B). Further linking the Vav1 SH3 domains to this response, we observed that the expression of the isolated Vav1 SH3-SH2-SH3 fragment also rescues the Vav1 deficiency in these cells (Figures 5B and S5A).

The rescue activity shown by the SH2 mutant version of Vav1 was rather unexpected because, to date, all known activities of Vav family proteins are SH2- and tyrosine phosphorylation-dependent (Bustelo, 2014). To further confirm these results, we decided to assess whether Vav1^WT^ could repress *HES1* promoter activity in Jurkat cells lacking surface expression of the TCRα/β (TCR^mut^). Vav1^WT^ is catalytically inactive in these cells as it cannot be tyrosine phosphorylated by upstream cytoplasmic protein tyrosine kinases (Barreira et al., 2014). We observed that Vav1^WT^ shows similar *HES1* promoter repression activity in both WT and TCR^mut^ cells (Figures 5C and S5B). Furthermore, it exhibits similar activity in unstimulated and TCR-stimulated WT Jurkat cells (Figure 5C). By contrast, the Vav1-mediated activation of the nuclear factor of stimulated T cells (NFAT) shows the expected dependence on TCR expression and stimulation status (Figures S5B and S5C). These results indicate that the Vav1-ICN1 connection can take place in a noncanonical SH2-, TCR-, and tyrosine phosphorylation-independent manner.

The Vav1 SH3 domains can bind to a large variety of proteins (Bustelo, 2014). However, based on our results indicating a connection of Vav1 with ubiquitinylation of ICN1 (Figure 4G), we suspected that one candidate for this regulatory step was the E3 ubiquitin ligase Cbl-b. This protein binds to the Vav1 C terminus via a canonical, proline-rich region (PRR)-mediated interaction (Bustelo et al., 1997). In favor of this possibility, we found that the shRNA-mediated *CBLB* knockdown in Jurkat cells phenocopies the effects of the Vav1 deficiency in terms of elevation of ICN1 abundance (Figures 5D and S5D) and ICN1 target gene expression (Figure 5E). These effects are not seen in Jurkat cells expressing either a scrambled shRNA or *CBLB* shRNAs that cannot abate Cbl-b expression (Figures 5D, 5E, and S5D). The expected changes in Cbl-b abundance in these cell lines were demonstrated using both immunoblots on Cbl-b immunoprecipitates (Figure 5D) and qRT-PCR (Figure S5E). These analyses also demonstrated that the effects induced by *CBLB* knockdown are not due to the reduction of *VAV1* transcript levels (Figure S5E). The alterations found in *CBLB* knockdown cells are due to the loss of the E3 ubiquitin ligase activity of Cbl-b, as they can be eliminated upon the ectopic expression of Cbl-b^WT^ but not of a catalysis-inefficient Cbl-b mutant (Y363F; Figures 5F, 5G, and S5F). Interestingly, the inhibitory effect induced by Cbl-b^WT^ on *HES1* promoter activity does not occur in *VAV1*^−/−^ Jurkat cells (Figures S5G and S5H), indicating that it is Vav1 dependent. Further confirming the implication of Cbl-b in this pathway, we observed that the depletion of Cbl-b leads to reductions in the amount of ICN1 ubiquitinylation similar to those triggered by Vav1 deficiency in Jurkat cells (Figures 5H and S5D). This pathway entails the formation of a trimeric complex of the endogenous proteins, as demonstrated by the detection of endogenous Vav1 and Cbl-b in ICN1 immunoprecipitates obtained from WT Jurkat cells (Figure 5I). These experiments also indicated that the coimmunoprecipitation (coIP) of the endogenous Cbl-b and ICN1 proteins is Vav1 dependent in those cells (Figure 5I).

### The Vav1 C-Terminal Region Nucleates the Formation of Cbl-B and ICN1 Complexes

**Figure 6:**
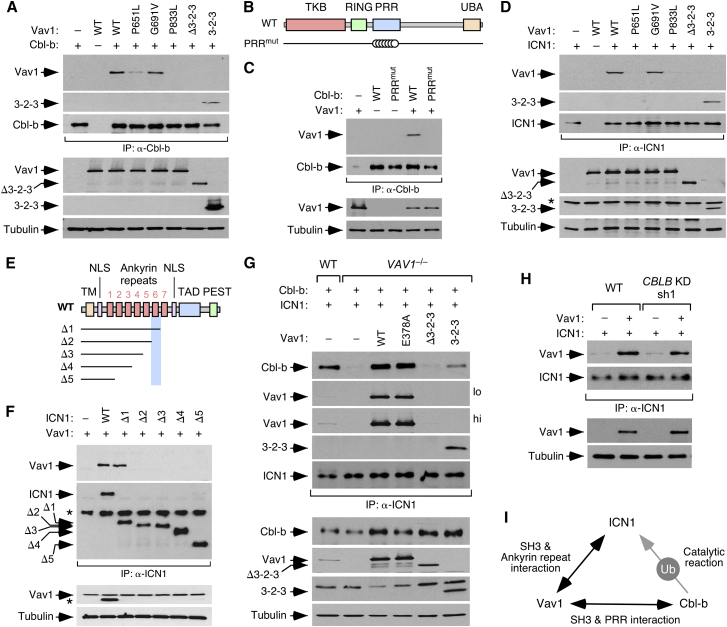
Structural Requirements for Vav1-Cbl-b-ICN1 Complex Formation (A) CoIP of Vav1 proteins with Cbl-b in Jurkat cells ectopically expressing the indicated combinations of proteins (top). Amount of immunoprecipitated Cbl-b was assessed by reblotting the same filter with antibodies to Cbl-b (third panel from top). Expression of ectopic Vav1 proteins (fourth and fifth panels from top) and endogenous tubulin α (loading control, bottom panel) was determined by WB using aliquots of the TCLs used in the immunoprecipitation step. (B) Depiction of the Cbl-b mutants used in these experiments. Mutations are shown as open circles. (C) CoIP of Vav1 with indicated Cbl-b proteins (top) in Jurkat cells. Controls for the immunoprecipitation and expression of proteins were done as indicated in (A). (D) CoIP of Vav1 proteins with ICN1 in Jurkat cells ectopically expressing the indicated combinations of proteins (top). Amount of immunoprecipitated ICN1 was assessed by reblotting the same filter with antibodies to ICN1. Expression of ectopic Vav1 proteins and endogenous tubulin α (loading control) was determined as in (A). Asterisk marks the tubulin α band from the previous immunoblot of the same filter. (E) Depiction of ICN1 mutants used in these experiments. TM, transmembrane; NLS, nuclear localization signal; TAD, transactivation domain. The domain whose deletion leads to loss of Vav1 binding is shaded in light blue. (F) CoIP of Vav1 with ICN1 mutant proteins in Jurkat cells expressing the indicated combinations of proteins (top). Controls for the immunoprecipitation and expression of proteins were done as indicated in previous panels. Asterisks in the second and third panels from the top indicate the immunoglobulin G band of the antibody to ICN1 and the ICN1 band remaining from the previous immunoblot of the same filter, respectively. (G) CoIP of Cbl-b and indicated Vav1 mutant proteins with ICN1 in WT and *VAV1*^−/−^ Jurkat cells. Controls for the immunoprecipitation and expression of proteins were done as indicated in previous panels. lo and hi refer to a low and a high exposure of the same film, respectively. (H) CoIP of Vav1 with ICN1 in WT and *CBLB* knockdown (clone #sh1) Jurkat cells. Controls for the immunoprecipitation and expression of proteins were done as in previous panels. (I) Summary of the interactions found in these experiments. Direct coIP and catalytic interactions are shown using black and gray arrows, respectively. Ub, ubiquitinylation. See also Figure S6.

We repeated the coIP experiments with ectopically expressed proteins to investigate the structural requirements involved in the formation of the Vav1-Cbl-b-ICN1 complex. We observed that the Vav1-Cbl-b interaction requires the functionality of the two Vav1 SH3 domains (Figures 6A and S6A) and the Cbl-b PRR (Figures 6B, 6C, and S6A). This interaction also occurs when Cbl-b is coexpressed with either the SH2 mutant version of Vav1 or the isolated SH3-SH2-SH3 fragment (Figures 6A and S6A). Vav1 exhibits the same structural requirements for the interaction with ICN1 (Figures 6D and S6A). All these results recapitulate the rescuing data obtained using the Vav1 mutant proteins in *VAV1*^−/−^ cells (Figure 5B). The use of ICN1 mutant proteins (Figure 6E) in coIP experiments revealed that the Vav1-ICN1 interaction requires the physical integrity of the sixth ICN1 ankyrin repeat but not of the most C-terminal domains (Figures 6F and S6A).

As in the case of the endogenous proteins (Figure 5I), the association of ICN1 and Cbl-b does not occur when those two proteins are ectopically expressed in *VAV1*^−/−^ Jurkat cells (Figures 6G and S6A). Binding can be rescued by expression of any Vav1 protein containing the SH3-SH2-SH3 cassette (Figures 6G and S6A). By contrast, we demonstrated using *CBLB*-knockdown Jurkat cells that the interaction of Vav1 with ICN1 is Cbl-b independent (Figures 6H and S6A). Taken together, these results indicate that Cbl-b and ICN1 utilize the Vav1 C terminus as a common docking platform to facilitate the subsequent ICN1 ubiquitinylation step (Figure 6I). These interactions must transiently occur in the cytoplasm because, unlike ICN1, endogenous Vav1 and Cbl-b are cytosolic in Jurkat cells (Figure S6B). Additional experiments with cells ectopically expressing both hemagglutinin (HA)-ubiquitin and ICN1 showed that ubiquitinylated ICN1 is located in the cytosol of Jurkat cells (Figure S6C).

### The Vav1-ICN1 Axis Is Downmodulated in Human TLX^+^ T-ALL

**Figure 7:**
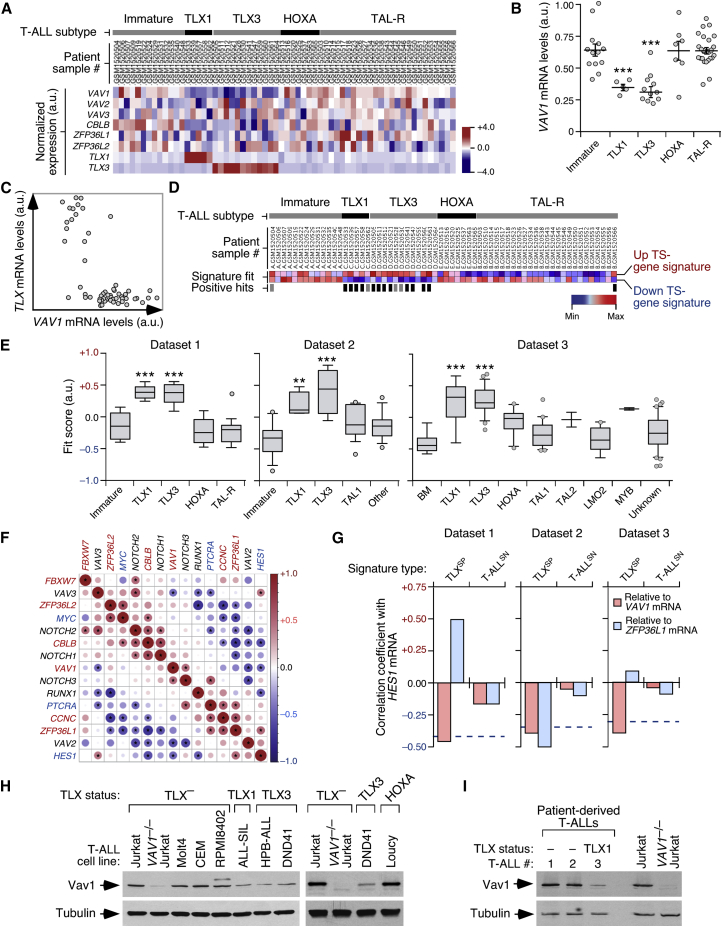
Vav1 Is Downmodulated in TLX^+^ T-ALL (A) Heatmap of indicated mRNAs (left) in T-ALL dataset 1. The identification number (left) and molecular subtype of patients (top) are indicated. Signal log ratio abundance is depicted as in Figure 2A. (B) Scatterplot showing *VAV1* expression across indicated human T-ALL subtypes (bottom) using microarray dataset 1. Dots represent values from an individual sample. Bars represent the mean expression value ± SEM for the overall sample set. ^∗∗∗^p ≤ 0.001 (Tukey's HSD test). (C) Scatterplot showing *VAV1* abundance against the combined amount of *TLX1/TLX3* expression using dataset 1. Dots represent values from individual samples. (D) ssGSEA-generated heatmap of the up- and downregulated genes of the “tumor-specific” *Vav1*^−/−^*/Zfp36l1*^−/−^;*Zfp36l2*^−/−^ signature in T-ALL cases from dataset 1. ssGSEA enrichment scores are depicted on a dark blue (lowest) to dark red (highest) scale. Samples with moderate and high signature fits are highlighted by gray and black bars, respectively. TS, tumor-specific. (E) Box plot of the tumor-specific *Vav1*^−/−^*/Zfp36l1*^−/−^;*Zfp36l2*^−/−^ gene signature fit score for indicated T-ALL subtype samples (bottom) and microarray datasets (top). Data are presented as indicated in Figure 2C. ^∗∗^p ≤ 0.01, ^∗∗∗^p ≤ 0.001 (Tukey's HSD test). (F) Expression correlation matrix from TLX^+^ T-ALL samples positive for the “tumor-specific” *Vav1*^−/−^*/Zfp36l1*^−/−^;*Zfp36l2*^−/−^ gene signature using dataset 1. Positive and negative correlation is shown in red and blue, respectively. The size of circles and color intensity are proportional to the Pearson correlation coefficient found for each transcript pair. Correlations with p values below the significance threshold of 0.05 (which relates with a Pearson correlation coefficient above 0.39 for dataset 1) have been considered as statistically significant and labeled with asterisks. Negative regulators of the Notch1 route and ICN1 targets are shown in red and blue letters, respectively. (G) Pearson correlation coefficient of the *HES1* mRNA with indicated transcripts (inset) and microarray datasets (top). The horizontal blue broken lines depict the p-value threshold used (0.05) to consider a correlation statistically significant. TLX^SP^, TLX^+^ T-ALL signature positive; T-ALL^SN^, T-ALL signature negative. (H and I) Expression of endogenous Vav1 in TCLs from indicated T-ALL cell lines (H) and patient-derived tumor cells (I) (n = 3). TLX and HOXA status of cells is shown on top. See also Figure S7.

Taking into consideration our genetic and signaling results, we surmised that the Vav1-Cbl-b axis could play tumor-suppressor roles in human T-ALL, namely those of the immature, TCR^–^ phenotype. This led us to investigate whether this pathway was either silenced or downregulated in specific T-ALL subtypes. Given that no mutations in *VAV1* and *CBLB* have been found in the T-ALL cases cataloged so far (http://cancer.sanger.ac.uk/cosmic), we decided to use an *in silico* approach to identify T-ALL patient subtypes that could fulfill three classification criteria: (1) low abundance of *VAV1* transcripts according to expression heatmap analyses; (2) overlap of the transcriptomes with a mouse *Vav1*^−/−^ T-ALL-associated gene signature (Table S3) according to both ssGSEA and GSEA; and (3) inverse relationship between the abundance of *VAV1* and *HES1* mRNAs (the latter one used as an indirect readout for Notch1 activity status) according to expression correlation matrices. For these analyses, we utilized three microarray datasets that included 240 patient samples representative of the molecular immature (mostly LYL1^+^), early cortical (mostly TLX1^+^, TLX3^+^ or HOXA^+^), and late cortical (TAL1^+^) T-ALL disease subtypes (Van Vlierberghe and Ferrando, 2012). These analyses indicated that the TLX^+^ T-ALL clinical subtype cases consistently showed a statistically significant reduction in the abundance of the *VAV1* mRNA (Figures 7A–7C, S7A, and S7B) as well as high transcriptional similarity to a diagnostic signature composed of gene probes commonly deregulated in *Vav1*^−/−^ and *Zfp36l1*^−/−^;*Zfp36l2*^−/−^ T-ALL cells (Figures S7C–S7E). The latter molecular feature is maintained even when using a more stringent gene signature (Table S3) in which gene probes directly associated with normal T cell differentiation were not included (Figures 7D, 7E, and S7F), thus ruling out that this cross-species similarity could be due to the arrest of mouse and human tumor cells in a common, “cortical”-like differentiation stage. The TLX^+^ T-ALL samples are also the only ones showing an inverse correlation between the abundance of *VAV1* and *HES1* transcripts (Figures 7F, 7G, and S7G). This is specific, since the abundance of the *HES1* mRNA shows no consistent inverse correlation with transcripts for other Vav family members, Cbl-b, and negative regulators of the Notch1 pathway such as the Zfp36L proteins, Fbxw7, and cyclin c (Figures 7F, 7G, and S7G). The only exception is the *ZFP36L1* transcript, whose abundance is inversely correlated with the amount of *HES1* mRNA in one out of the three interrogated datasets (Figure 7G, dataset #2). Reinforcing these *in silico* data, we observed that the abundance of endogenous Vav1 is consistently lower in TLX^+^ than in TLX^–^ T-ALL cell lines (Figures 7H and S7H). More importantly, Vav1 levels are also lower in tumor cells from a TLX1^+^ T-ALL patient than in two TLX^–^ T-ALL patient-derived samples (Figures 7I and S7H).

### The TLX-Mediated Downmodulation of Vav1 Is Required for T-ALL Pathogenesis

**Figure 8:**
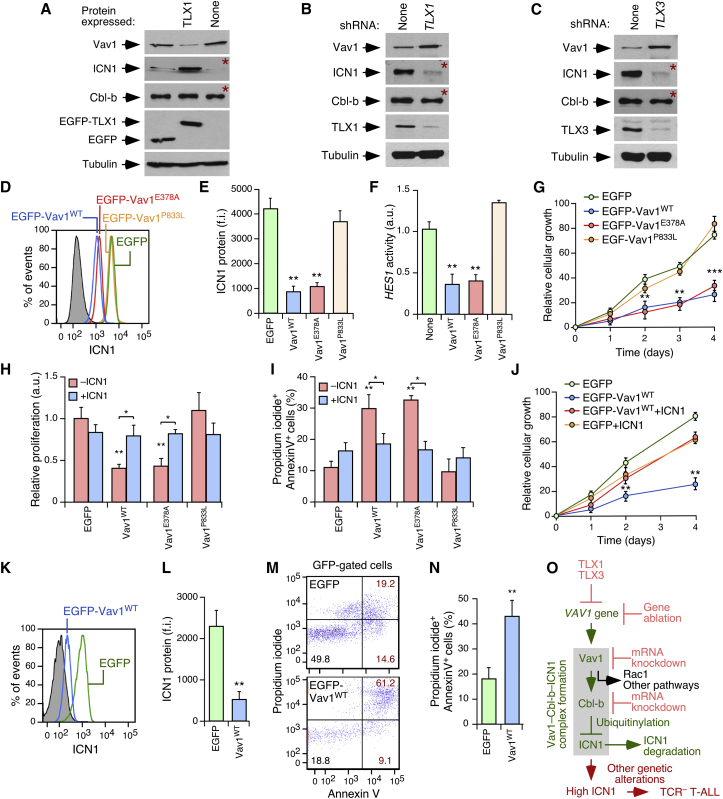
The TLX-Mediated Downmodulation of Vav1 Is Important for TLX^+^ T-ALL Pathogenesis (A) Effect of ectopic expression of EGFP-TLX1 in the abundance of Vav1, ICN1 and Cbl-b in Jurkat cells. Detection of EGFP-TLX1 and EGFP control was carried out using antibodies to GFP (fourth panel from top). (B) Effect of *TLX1* knockdown in the abundance of Vav1, ICN1 and Cbl-b in ALL-SIL cells. (C) Effect of *TLX3* knockdown in the abundance of Vav1, ICN1 and Cbl-b in HPB-ALL cells. Red asterisks indicate panels generated using electrophoresed TCLs transferred to an independent nitrocellulose filter. (D and E) Example (D) and quantification (E) of the effect of the ectopic expression of indicated EGFP-Vav1 proteins in ICN1 abundance in HPB-ALL cells (n = 3). (F) Effect of indicated Vav1 proteins on *HES1* promoter activity (n = 3). (G) Effect of indicated EGFP-Vav1 proteins in the growth of HPB-ALL cells (n = 3). (H and I) Effect of indicated EGFP-Vav1 proteins in the proliferation (H) and apoptosis (I) of HBP-ALL cells in the absence or presence of ectopically expressed ICN1 (n = 3). (J) Effect of the expression of EGFP-Vav1^WT^ and ICN1 in the growth of HPB-ALL cells (n = 3). (K and L) Example (K) and quantification (L) of the effect of EGFP-Vav1^WT^ on ICN1 levels in tumor cells from a TLX1^+^ T-ALL patient (n = 3). (M and N) Example (M) and quantification (N) of the effect of EGFP-Vav1^WT^ in the apoptosis of tumor cells from a TLX1^+^ patient (n = 3). (O) The pathway unveiled in this work. The Vav1 suppressor and canonical routes are shown in green and black, respectively. The Vav1-Cbl-b-ICN1 complex is depicted as a gray box. Disease and experimental conditions disrupting this signaling axis are in red. Data shown in (E–J), (L), and (N) represent mean ± SEM. ^∗^p ≤ 0.05, ^∗∗^p ≤ 0.01, ^∗∗∗^p ≤ 0.001 (Student's t test). See also Figure S8.

The previous results suggested that one of the potential functions of the TLX transcriptional repressor family is to downregulate the Vav1-Cbl-b pathway in TLX^+^ T-ALL cells. Consistent with this hypothesis, *in silico* analyses of chromatin immunoprecipitation sequencing (ChIP-seq) and ChIP-chip data revealed that *VAV1*, but not *VAV2*, has binding sites for TLX1, TLX3, and the associated transcriptional corepressor ETS1 (Figure S8A). Furthermore, we observed that the ectopic expression of TLX1 in the TLX^–^ Jurkat cell line leads to reductions in Vav1 abundance and the concurrent upregulation of ICN1 (Figures 8A and S8B). The ectopic expression of TLX1, however, does not induce any significant increase in the already high levels of ICN1 present in *VAV1*^−/−^ Jurkat cells (Figures S8B and S8C), further indicating that the effect of TLX1 on ICN1 abundance in WT cells is directly linked to the repression of *VAV1*. Conversely, we found that knockdown of endogenous *TLX1* in TLX1^+^ ALL-SIL cells promotes expression of endogenous Vav1 and reduces ICN1 (Figures 8B and S8B). Similar data were obtained when *TLX3* was knocked down in TLX3^+^ HPB-ALL cells (Figures 8C and S8B). The manipulation of TLX levels does not have any significant impact on the abundance of Cbl-b in any of those cells (Figures 8A–8C and S8B).

We next investigated the effect of the forced expression of Vav1 in the pathological features of TLX^+^ and TLX^–^ T-ALL cell lines. We found that the ectopic expression of Vav1^WT^ leads to decreased ICN1 levels (Figures 8D, 8E, and S8D), reduced *HES1* promoter activity (Figures 8F and S8E), reduced proliferation (Figures 8G, 8H, and S8F), and increased apoptosis (Figures 8I and S8F) in HPB-ALL cells. The same effects can also be elicited by a catalytically dead Vav1 protein (E378A) but not by a Cbl-b- and ICN1-binding defective CSH3 (P833L) mutant counterpart (Figures 8D–8I and S8D–S8F). The coexpression of ICN1 eliminates the negative effects induced by ectopically expressed Vav1 on both the proliferation and viability of HPB-ALL cells (Figures 8H–8J and S8F), further indicating that they are the consequence of reduced ICN1 signaling. The ectopic expression of Vav1^WT^ also results in reduced abundance of ICN1 as well as the proliferation and survival of ALL-SIL (Figures S8G–S8J) and TLX1^+^ patient-derived T-ALL (Figures 8K–8N) cells. By contrast, we did not observe any significant impact of overexpressed Vav1^WT^ on the proliferation and/or survival of the TLX^–^ Loucy (which do not express ICN1) (O'Neil et al., 2007), Jurkat, and Molt4 T-ALL cells (Figures S8K–S8M). These results underscore the importance of the downregulation of the Vav1-Cbl-b-Notch1 axis to maintain TLX^+^ T-ALL pathogenesis (Figure 8O).

## Discussion

Rho GEFs in general, and Vav proteins in particular, have been traditionally linked to protumorigenic pathways (Zandvakili et al., 2017). Despite this evidence, the roles played by these proteins in cancer cells remain poorly characterized. Our work indicates that these functions can be more variegated than previously anticipated. Thus, we have shown that Vav1 can play a GTPase-independent, tumor-suppressor-like role that mediates Notch1 signaling in T cells. This pathway seems to act by default in immature thymocytes and many T-ALL cells, as determined by the rapid changes in ICN1 abundance recorded when the amounts of either Vav1 or Cbl-b are genetically manipulated. It also seems independent of the mutational status of *NOTCH1* and *FBXW7*, since Vav1 and Cbl-b can alter ICN1 levels even in cells bearing mutant alleles in both loci. Vav1 participates in this pathway in an SH2 domain- and protein tyrosine kinase-independent manner, indicating that it follows regulatory models different from the archetypical, phosphorylation-mediated stimulation of the canonical Vav1-dependent pathways (Bustelo, 2014). This might explain the need for the transcriptional regulation of the gene in TLX^+^ T-ALL cells, a method of modulation seldom found in Vav1.

The knockout of *Vav1* in mice leads to unbalanced Notch1 signaling in immature T cells and the rapid emergence of T-ALL. This transformation step requires additional lesions in other unidentified loci, a result consistent with previous observations indicating that Notch1 signaling elements are not leukemogenic per se unless combined with other predisposing genetic alterations (Chiang et al., 2008). It is likely that this is also the reason for the longer latencies required for the development of spontaneous T cell tumors previously described in aging *Vav1*^−/−^ mice (Ruiz et al., 2009). All leukemias detected in *Vav1*^−/−^ mice consistently lack membrane expression of the TCR and are arrested at either the DN or ISP developmental stages. This feature is consistent with both the developmental boundaries of Notch1 signaling in thymocytes and the immunophenotype of T-ALLs that develop in *HES1* transgenic mice (Dudley et al., 2009, Xiong et al., 2011). However, it is at odds with the frequent detection of TCR^+^ tumor cells in both patients and mouse models of Notch1-driven leukemogenesis (O'Neil et al., 2006, Van Vlierberghe and Ferrando, 2012). A possible interpretation for these findings is that the transformed *Vav1*^−/−^ cells cannot overcome the lack of signals required for the ISP to CD4^+^CD8^+^ transition and/or for Notch1-driven transformation. Alternatively, it might reflect a higher level of dependency of TCR^+^ T-ALL on the canonical pathways regulated by Vav1 that are lost in the case of *Vav1*^−/−^ mice. This latter model would also explain the detection of *VAV1* gain-of-function mutations in tumors representative of mature T cell stages. This type of bivalent, developmental window-associated tumor-suppressor and promoting programs has been demonstrated before for the transcriptional factor Runx1 in TCR^–^ and TCR^+^ T-ALL (Della Gatta et al., 2012, Sanda et al., 2012).

The downregulation of this Vav1-dependent pathway, via transcriptional repression of the *VAV1* rather than loss-of-function mutations, seems to represent a key contributing factor in the pathogenesis of human TLX^+^ T-ALL. This inhibition is mediated by the direct targeting of *VAV1* regulatory sequences by the TLX repressor complex. This action does not totally silence *VAV1* expression, suggesting that some residual Vav1 activity could still be required to favor, for example, some basal level of stimulation of its catalysis-regulated routes. Alternatively, it might simply reflect the fact that the total depletion of Vav1 is not required to achieve a significant upregulation of ICN1 signaling. This latter idea is consistent with the variations in ICN1 levels and activity observed in T-ALL cells in which the abundance of endogenous Vav1 was manipulated by either expressing or depleting TLX and Vav1. Interestingly, a recent report has shown that TLX proteins have a rather ambivalent and tumorigenic phase-specific relationship with the Notch1 pathway (Durinck et al., 2015). According to this model, TLX proteins repress the expression of ICN1-regulated genes during the preneoplasic phase to possibly facilitate a thymocyte developmental arrest in an immature, TCR^–^ stage. Subsequently, a hyperactivation of ICN1 signaling seems to be required to bypass this preneoplasic phase and develop the disease. Although not formally corroborated *in vivo*, this model is interesting because it provides a rational explanation for the high frequency of mutations in the Notch1 pathway typically found in this T-ALL subtype (Neumann et al., 2015). Assuming this model, we could interpret the elimination of the Vav1-Cbl-b-ICN1 axis in this latter phase as a way to ensure higher levels of ICN1 signaling in fully developed T-ALL cells irrespective of the mutational status of the Notch1 pathway. However, the inhibition of the Vav1-Cbl-b axis in the preneoplasic phase seems at odds with the theoretical need of the TLX-mediated repression of ICN1 target genes that must take place at that stage. One possible explanation for this contradiction is that the upregulation of ICN1, whose activity can be counteracted further downstream by the direct repression of its target genes by TLX, can represent a necessary toll that the transcriptional factor has to pay to make possible the elimination of other Vav1-dependent functions that could be more important for this pretumorigenic stage. For example, it is possible that the elimination of the canonical, Vav1 GEF-dependent pathways could contribute to further amplify or secure the TLX-mediated developmental arrest of thymocytes. This hypothesis is consistent with the known roles of the Vav1-Rac1 pathway in thymocyte development (Bustelo, 2014). In line with this, preliminary results indicate that *Vav1*^−/−^ and TLX1-transduced primary DN thymocytes show a similar developmental arrest when cultured *ex vivo* in the presence of Delta1-expressing OP9 cells (J.R.-V. and X.R.B., unpublished data). Although our evidence indicates that the mechanistic basis of the tumor-suppressor activity of Vav1 is probably the same in mouse and human systems, it is worth noting that the leukemia that develops in *Vav1*^−/−^ mice is different from TLX-driven T-ALL in a number of features, including the lack of frequent concurrent mutations in *Notch1*, *Fbxw7*, and *Pten*. It would be interesting to sequence the genome of *Vav1*^−/−^ T-ALL to identify the gene lesions that cooperate in this leukemogenic process and, at the same time, to verify whether they could include the loss of other tumor-suppressor genes commonly found in TLX^+^ T-ALL that had not been explored in the present work.

Could this Vav1 suppressor pathway be active in other Notch family-driven tumors? Available genetic evidence suggests that this could be the case, since some of the *VAV1* mutations found in both peripheral T cell leukemia and lung tumors generate C-terminally truncated proteins that cannot interact with Cbl-b. These mutant proteins can be divided into catalytically hyperactive and deficient subsets (J.R.-V. and X.R.B., unpublished data), suggesting that the Vav1 tumor-suppressor and protumorigenic activities can become deregulated independently or concurrently in a tumor- and patient-specific manner. Further work is needed, however, to determine the actual connection of these mutant subgroups with Notch1 signaling and clinically relevant features of cancer patients.

This GTPase-independent Vav1 suppressor pathway will be preserved in cancer cells even under conditions of inhibition of Vav1 catalytic activity. This is therapeutically interesting because, to date, the targeting of the catalytic domains of Rho GEFs is believed to be the most feasible strategy for drug development in the field (Vigil et al., 2010). By contrast, our results caution against the use of Vav1 depletion avenues, especially in the case of Vav1-dependent tumors positively regulated by Notch1 signals. They also challenge the widely assumed paradigm that links the actions of Rho GEFs with protumorigenic effects in cancer cells, further underscoring the need for the comprehensive characterization of this family using genetic and animal models.

## STAR★Methods

### Key Resources Table

**Table:** 

REAGENT or RESOURCE	SOURCE	IDENTIFIER
**Antibodies**		

FITC Rat Anti-Mouse CD4, Clone GK1.5	BD Biosciences	Cat# 553729 RRID: AB_395013
APC Rat Anti-Mouse CD4, Clone RM4-5	BD Biosciences	Cat# 553051 RRID: AB_398528
APC-H7 Rat Anti-Mouse CD4, Clone GK1.5	BD Biosciences	Cat# 560181 RRID:AB_1645235
V500 Rat Anti-Mouse CD4, Clone RM4-5	BD Biosciences	Cat# 560783 RRID:AB_1937327
FITC Rat Anti-Mouse CD8a, Clone 53-6.7	BD Biosciences	Cat# 553031 RRID: AB_394569
Pacific Blue™ Rat Anti-Mouse CD8a, Clone 53 6.7	BD Biosciences	Cat# 558106 RRID: AB_397026
PE Rat Anti-Mouse CD8a, Clone 53-6.7	BD Biosciences	Cat# 553032 RRID:AB_2034011
APC Rat Anti-Mouse CD25, Clone 3C7	BD Biosciences	Cat# 558643 RRID:AB_1645222
PE-Cy™7 Rat Anti-Mouse CD25, Clone PC61	BD Biosciences	Cat# 561038 RRID:AB_2034002
APC Anti-Mouse CD24 Monoclonal Antibody, Clone M1/69	eBioscience	Cat# 17-0242-82 RRID:AB_10870773
PerCP-Cyanine5.5 Anti-Mouse CD45R (B220) Monoclonal Antibody, Clone RA3-6B2	eBioscience	Cat# 45-0452-82 RRID:AB_1107006
PerCP-Cy™5.5 Rat Anti-Mouse CD44, Clone IM7	BD Biosciences	Cat# 560570 RRID:AB_1727486
PE Anti-Mouse TCR beta Monoclonal Antibody, Clone H57-597	eBioscience	Cat# 12-5961-82 RRID:AB_466066
PE-Cyanine7 Anti-Human CD5 Monoclonal Antibody, Clone UCHT2	eBioscience	Cat# 25-0059-42 RRID:AB_1582282
PE Anti-Human CD7 Monoclonal Antibody, Clone 4H9	eBioscience	Cat# 12-0078-41 RRID:AB_2572549
APC Anti-Human CD45 Monoclonal Antibody, Clone HI30	eBioscience	Cat# 17-0459-41 RRID:AB_10671389
Cleaved Notch1 (Val1744), Clone D3B8, Rabbit mAb	Cell Signaling Technology	Cat# 4147S RRID:AB_2153348
PE Anti-Notch1 Mouse Monoclonal Antibody, Clone mN1A	eBioscience	Cat# 552768 RRID:AB_394454
Anti-α-Tubulin Mouse mAb, Clone DM1A	Calbiochem	Cat# CP06 RRID:AB_2617116
Anti-Vav1 Rabbit (DH domain)	Homemade	N/A
Anti-Vav1 Rabbit (SH2 domain)	Homemade	N/A
Purified Anti-HA.11 Epitope Tag Antibody, Clone 16B12	Covance	Cat# MMS-101P RRID:AB_2565018
Anti-Cbl-b Rabbit mAb, Clone D3C12	Cell Signaling Technology	Cat# 9498S
Purified Anti-GFP Epitope Tag Antibody, Clone B34	Covance	Cat# MMS-118P RRID:AB_2565021
Anti-CD3 Antibody, Clone UCHT1	Millipore	Cat# CBL150 RRID:AB_93225
Anti-Activated Notch1 Polyclonal Antibody	Abcam	Cat# ab52301 RRID:AB_881726
Anti-Notch1 (C-20) antibody	Santa Cruz Biotechnology	Cat# sc-6014 RRID:AB_650336
Anti-Human CD98 Antibody, Clone EPR3548(2)	Abcam	Cat# ab108300 RRID:AB_2190677
Anti-Human PCNA Antibody, Clone EPR3821	Abcam	Cat# ab92552 RRID:AB_10561784
Anti-GlyRS Rabbit Polyclonal Antibody, Unconjugated, Clone H300	Santa Cruz Biotechnology	Cat# sc-98614 RRID:AB_2107783
Anti-Hox11/TLX1 Antibody, Clone C-18	Santa Cruz Biotechnology	Cat# sc-880 RRID:AB_2203789
Anti-TLX3 Antibody, Clone 34-L	Santa Cruz Biotechnology	Cat# sc-81990 RRID:AB_1130420

**Bacterial and Virus Strains**		

Dh5-Alpha Competent *E*. *coli*	Life Technologies	Cat# 18258012

**Biological Samples**		

Patient-derived T-ALL Xantal C-n° 240	Dr. M. Camós Lab	N/A
Patient-derived T-ALL 1	Dr. M.L. Toribio Lab	N/A
Patient-derived T-ALL 9	Dr. M.L. Toribio Lab	N/A

**Chemicals, Peptides, and Recombinant Proteins**		

7,12-dimethyl-α-benzanthracene (DMBA)	Sigma	Cat# D3254
Urethane	Sigma	Cat# U2500
Methylnitrosourea (MNU)	Sigma	Cat# N1517
BD Cytofix/Cytoperm™	BD Biosciences	Cat# 554714
Trizol	Sigma	Cat# T9424
Compound E (gamma-secretase inhibitor)	Alexis Biochemicals	Cat# ALX-270-415
DAPT (gamma-secretase inhibitor)	Alexis Biochemicals	Cat# ALX-270-416
MG132	Calbiochem	Cat# 474790
GammaBind™ G Sepharose™	GE Healthcare	Cat# GE17-0885-01
Ivermectin	Sigma	Cat# I8898
Puromycin	Sigma	Cat# P9620
γ-Secretase Substrate, Fluorogenic	Calbiochem	Cat# 565764

**Critical Commercial Assays**		

QuikChange mutagenesis kit II	Agilent Technologies	Cat# 200522
iScript One-Step RT-PCR kit with Syber Green	BioRad	Cat# 170-8893
Dual-Luciferase Reporter Assay System	Promega	Cat# E1960
Neon™ Transfection System 100 μL Kit	ThermoFisher Scientific	Cat# MPK10096
Annexin V–fluorescein propidium isothiocyanate detection kit	Immunostep	Cat# ANXVKF-100T

**Deposited Data**		

Microarray data	This study	GEO: GSE80490

**Experimental Models: Cell Lines**		

Mouse OP9-GFP	Dr. M.L. Toribio Lab	N/A
Mouse OP9-DL1	Dr. M.L. Toribio Lab	N/A
Human WT Jurkat E6.1	ATCC	Cat# TIB-152 RRID:CVCL_0367
Human *Vav1*^*–/–*^ Jurkat	Dr. R.T. Abraham Lab	N/A
Human Vav1^WT^-reconstituted Jurkat	Dr. R.T. Abraham Lab	N/A
Human CEM	Dr. A. Bigas Lab	N/A
Human CEM-KO Vav1 Clone 1	This study	N/A
Human CEM-KO Vav1 Clone 3	This study	N/A
Human Molt4	Dr. A. Bigas Lab	N/A
Human Molt4-KO Vav1 Clone 1	This study	N/A
Human Molt4-KO Vav1 Clone 3	This study	N/A
Human WT Jurkat-KO Vav1 Clone 1	This study	N/A
Human WT Jurkat-KO Vav1 Clone 2	This study	N/A
Human WT Jurkat-KO Vav1 Clone 3	This study	N/A
Human Jurkat J.31.13 (TCR^mut^)	Dr. B. Alarcón Lab	N/A
Human WT Jurkat-KO Cbl-b Clone 1	This study	N/A
Human WT Jurkat-KO Cbl-b Clone 2	This study	N/A
Human WT Jurkat-KO Cbl-b Clone 3	This study	N/A
Human WT Jurkat-KO Cbl-b Clone 4	This study	N/A
Human RPMI 8402	Dr. A. Bigas Lab	N/A
Human ALL-SIL	DMSZ	Cat# ACC-511 RRID:CVCL_1805
Human HPB-ALL	Dr. A. Bigas Lab	N/A
Human DND41	Dr. A. Bigas Lab	N/A
Human Loucy	Dr. A. Bigas Lab	N/A
Human ALL-SIL-KO TLX1	This study	N/A
Human HPB-ALL-KO TLX3	This study	N/A

**Experimental Models: Organisms/Strains**		

Mouse: *Vav1*^*–/–*^ (C57BL/10)	(Turner et al., 1997)	N/A
Mouse: *Vav1*^*–/–*^; *Vav2*^*–/–*^; *Vav3*^*–/–*^ (C57BL/10)	(Fabbiano et al., 2015)	N/A
Mouse: NOD-*scid IL2rgnull*	The Jackson Laboratory	Cat# 005557

**Oligonucleotides**		

See Table S4 for Primers	This study	N/A

**Recombinant DNA**		

pEF1α/Myc-HisA	ThermoFisher Scientific	Cat# V92120
Mouse Vav1^WT^ (pJLZ52)	(Zugaza et al., 2002)	N/A
Mouse Vav1^E378A^ (pJRV29)	This study	N/A
Mouse Vav1^P651L^ (pKES46)	(Zugaza et al., 2002)	N/A
Mouse Vav1^G691^ (pKES35)	(Zugaza et al., 2002)	N/A
Mouse Vav1^P833L^ (pKES42)	(Zugaza et al., 2002)	N/A
Mouse Vav1^Δ3-2-3^ (pKES17)	(Zugaza et al., 2002)	N/A
Mouse Vav1 SH3-SH2-SH3 (pSRM22)	(Barreira et al., 2014)	N/A
pEGFP-C2	Clontech	Cat# 632481
Mouse EGFP-Vav1^WT^ (pSRM3)	(Barreira et al., 2014)	N/A
Mouse EGFP-Vav1^E378A^ (pJRV32)	This study	N/A
Mouse EGFP-Vav1^L334A+R375A^ (pJRV02)	This study	N/A
Mouse EGFP-Vav1^P651L^ (pMB50)	(Barreira et al., 2014)	N/A
Mouse EGFP-Vav1^G691V^ (pMB51)	(Barreira et al., 2014)	N/A
Mouse EGFP-Vav1^P833L^ (pMB68)	(Barreira et al., 2014)	N/A
Mouse EGFP-Vav1^Δ3-2-3^ (pNM114)	(Zugaza et al., 2002)	N/A
Mouse EGFP-Vav1 SH3-SH2-SH3 (pNM117)	(Zugaza et al., 2002)	N/A
Human Cbl-b (pXRB118)	(Bustelo et al., 1997)	N/A
Human Cbl-b^Y363F^ (pJRV19)	This study	N/A
Human Cbl-b^PRRmut^ (pJRV41)	This study	N/A
Mouse ICN1 (pICN1)	(Espinosa et al., 2003)	N/A
Mouse ICN1^ΔANK7-2531^ (pJRV44) (Δ1 in Figure 6E)	This study	N/A
Mouse ICN1^ΔANK6-2531^ (pJRV54) (Δ2 in Figure 6E)	This study	N/A
Mouse ICN1^ΔANK5-2531^ (pJRV53) (Δ3 in Figure 6E)	This study	N/A
Mouse ICN1^ΔANK4-2531^ (pJRV42) (Δ4 in Figure 6E)	This study	N/A
Mouse ICN1^ΔANK2-2531^ (pJRV43) (Δ5 in Figure 6E)	This study	N/A
Mouse ICN1^A2060V^ (pJRV55)	This study	N/A
pSSK-HA-ubiquitin (pHA-Ub)	Dr. M. Pagano Lab	N/A
pNFAT-Luc	Dr. G.R. Crabtree Lab	N/A
pCBF1-Luc	(Espinosa et al., 2003)	N/A
pHES1-Luc	(Espinosa et al., 2003)	N/A
pRL-SV40 (Renilla vector)	Promega	Cat# E2231
pMSCV-GFP retroviral vector	Dr. A. Ferrando Lab	N/A
pMSCV-GFP-TLX1 retroviral vector	Dr. A. Ferrando Lab	N/A
pHRSIN lentiviral vector	Dr. A. Rodríguez Lab	N/A
Mouse EGFP-Vav1^WT^ lentiviral vector (pJRV27)	This study	N/A

**Software and Algorithms**		

FlowJo (version 8.7.3)	FlowJo, LLC	https://www.flowjo.com/solutions/flowjo
R	R Core Team	https://www.R-project.org/
RMA	Bioconductor	http://bioconductor.org/packages/release/bioc/html/affy.html
Limma	Bioconductor	https://bioconductor.org/packages/release/bioc/html/limma.html
DAVID	DAVID website	https://david.ncifcrf.gov
Heatmap3	CRAN	http://CRAN.R-project.org/package=heatmap3
Corrplot	CRAN	http://CRAN.R-project.org/package=corrplot
GSEA	Broad Institute	http://software.broadinstitute.org/gsea/index.jsp
ssGSEA	GenePattern	https://genepattern.broadinstitute.org/gp/pages/login.jsf
Bowtie	Bowtie website	http://bowtie-bio.sourceforge.net/index.shtml
MACS1.4	Python	https://pypi.python.org/pypi/MACS
LiftOver	UCSC	http://genome.ucsc.edu/goldenPath/help/hg18ToHg19LiftOver.html
wig2bed	GitHub	https://github.com/bedops/bedops/blob/master/applications/bed/conversion/src/wrappers/wig2bed
Gviz	Bioconductor	https://bioconductor.org/packages/release/bioc/html/Gviz.html
StepOne software (version 2.1)	ThermoFisher Scientific	https://www.thermofisher.com/order/catalog/product/4376600
ImageJ (version 1.44p)	NIH Image	https://imagej.nih.gov/ij/
GraphPad Prism (version 6.0)	GraphPad Software Inc	https://www.graphpad.com/scientific-software/prism/
Other		
Micro-osmotic pumps, Model 1002	Alzet	Cat# 004317

### Contact for Reagent and Resource Sharing

Further information and requests for resources and reagents should be directed to, and will be fulfilled by, the Lead Contact Xosé R. Bustelo (xbustelo@usal.es).

### Experimental Model and Subject Details

#### Mouse Experiments

Animal work was done according to protocols approved by the Bioethics committee of Salamanca University. *Vav1*^–/–^ and *Vav1*^–/–^;*Vav2*^–/–^;*Vav3*^–/–^ mice have been previously described (Fabbiano et al., 2015, Turner et al., 1997). All mouse strains used were homogenized to the C57BL/10 genetic background. In the case of chemical carcinogenic experiments, DMBA (Cat. No. D3254; Sigma) was dissolved in cottonseed oil at a concentration of 10 mg/ml and administered (0.1 ml) weekly via intragastric intubation to female mice of indicated genotypes for a total of six weeks (Figure 1A). The first administration started when animals were 8-week-old. In the case of N-nitroso-N-methylurea (Cat. No. N1517, Sigma), a single intraperitoneal injection was administered (50 mg/kg of body weight) to 5-week-old animals. In the case of urethane (1 g/kg of body weight, Cat. No. U2500, Sigma), 8-week-old mice of indicated genotypes were intraperitoneally injected for a total of six weeks. Mice were then examined weekly until showing obvious physical signs of sickness. Upon euthanasia, the indicated tissues and peripheral blood were collected for histological processing, flow cytometry analyses, and extraction of either total cellular proteins or RNAs. In the case of DAPT treatments, DMBA-treated *Vav1*^–/–^ mice showing a leukemic ISP status according to flow cytometry determinations of peripheral blood were treated with DAPT (4 mg/kg of body weight/day; Cat. No. ALX-270-416; Alexis Biochemicals) for 28 days using osmotic delivery pumps (Model 1002; Alzet) subcutaneously implanted in the backs of animals. For all *in vivo* studies, female animals of the same genotype were randomly assigned to the different experimental groups. No animal was discarded for the final evaluation of results.

#### Primary Mouse Tumor Cells

Leukemic cells from *Vav1*^–/–^ mice were cocultured with feeder layers of OP9 stromal cells overexpressing either GFP (OP9-GFP) or the Delta-like 1 protein (OP9-DL1) in MEMα supplemented with 20% fetal calf serum. OP9 cells were provided by Dr. M.L. Toribio. When indicated, cells were treated with either Compound E (200 nM, Enzo) or vehicle alone (DMSO) and collected at the indicated time points. For *in vivo* experiments, 500,000 cells were intravenously injected into recipient WT mice.

#### Cell Lines

WT Jurkat cells were obtained from the ATCC. *VAV1*^–/–^ and Vav1^WT^-reconstituted Jurkat cells were provided by Dr. R.T. Abraham (Duke University Medical Center, Durham, NC) (Cao et al., 2002). Jurkat J.31.13 (TCR^mut^) cells were provided by Dr. B. Alarcón and described elsewhere (Alcover et al., 1988). Molt4, CEM, RPMI8402, HPB-ALL, DND41 and Loucy cells were provided by one of the authors of this study (A.B.). ALL-SIL cells were from the DSMZ cell line repository. T-ALL cell lines were cultured in RPMI1640 supplemented with 10% fetal calf serum. In the case of Jurkat cells, they were treated in some cases with antibodies to human CD3 (Cat. No. 217570, Calbiochem) or Compound E as above to stimulate them and inhibit Notch1 cleavage, respectively.

#### Patient-Derived T-ALL Cells

The use of patient samples was done according to methods and informed patient consent policies approved by the Bioethics committee of Hospital Sant Joan de Déu. Primary T-ALL cells of the TLX1^+^ (designated at the time of collection as Xantal C-#240) and TLX^–^ (#1 and #2, designated at the time of collection as TALL1 and TALL9, respectively) subtypes were initially obtained at M. Camós’ and M.L. Toribio’s labs, respectively. Cells were processed for genetic and flow cytometry characterization and rapidly stored in liquid nitrogen. For expansion, cells were thawed, cultured in the presence of feeder layers of OP9-DL1 cells in MEMα containing IL-7 (5 ng/mL, Peprotech), Flt3L (5 ng/mL, Peprotech) and 20% fetal calf serum for 48 hr, and injected into sublethally-irradiated (2 Gy) 6- to 8-week-old NOD-*Scid IL2rgnull* mice (NSG, Jackson Laboratory). Engrafted T-ALL blasts (CD5^+^CD7^+^CD45^+^) were collected by preparative flow cytometry from the thymi, spleens, and bone marrows of the recipient mice 10 weeks later. Cells were then frozen in liquid nitrogen and, when needed, cultured on OP9-DL1 cells as indicated above. The TLX1^+^ T-ALL cells (t(10;14)(q24;q11)[8]/46XX[17] carrying WT *NOTCH1* alleles) were TCRα/β^–^, TCRγ/δ^–^, CD45^++^, CD34^–^, TdT^+^ (58%), icCD3^+^, mCD3^low^, CD7^++^, CD5^++^, CD2^+^ (78%), CD4^+^, CD8^–^, CD1a^+^ (90%), CD10^+^, CD13^–^, CD33^–^, CD56^–^, CD123^–^, and CD117^–^. The TLX^–^ T-ALL cells were either mTCRα/β^+^ (100%), CD4^+^ (31%), CD8^–^, CD5^+^, CD7^+^, CD45^+^, IL7R^–^, ICN1^–^, PTEN^–^, and TLX^–^ (in the case of T-ALL #1) or mTCRα/β^+^ (10-30%), CD4^+^/CD8^+^ (76-90%), CD5^+^, CD7^+^, CD45^+^, ICN1^++^, PTEN^+^, and TLX^–^ (in the case of T-ALL #2). In addition to the cytogenetic analyses, the TLX status of primary tumor cells was confirmed by qRT-PCR both before and after expansion in immunocompromised mice. For lentiviral infections, thawed cells were cultured for 24 hr as above, infected with lentiviruses encoding either EGFP or the indicated EGFP-Vav1 proteins, centrifuged at 1800 rpm for 90 min without brake at room temperature, and maintained in the above media for 48 hr.

### Method Details

#### Construction of Expression Vectors

To generate the lentiviral vector encoding EGFP-tagged Vav1^WT^ (pJRV27), the *Vav1* cDNA was PCR amplified using the pJLZ52 plasmid as template and the oligonucleotide primers 5’-ATA GGAT CCG CCA CCA TGG AGC TCT GGC GAC AGT GCA CC-3’ (forward; BamHI site underlined) and 5’-AGC TAC TCG AGA ATA TTC AGT TAG AAG GGA ACC AGC C-3’ (reverse; XhoI site underlined), digested with BamHI and XhoI, and ligated into the pHRSIN vector (provided by A. Rodríguez, Department of Molecular Biology, Madrid Autonomous University, Madrid, Spain). Vectors encoding additional Vav1, Cbl-b and ICN1 mutant proteins are listed in Key Resources Table. Mutations were generated by in situ mutagenesis using the QuikChange mutagenesis kit II (Agilent Technologies). Oligonucleotides used for the mutagenesis steps are listed in Table S4. All newly generated plasmids were subjected to DNA sequence analysis to confirm both the generation of the proper mutation and the absence of unwanted ones.

#### Histology

Tissues were fixed in 4% paraformaldehyde in phosphate-buffered saline solution, paraffin-embedded, cut in 2-3 μm sections and stained with hematoxylin and eosin (Sigma). Sections were blindly analyzed by an independent pathologist.

#### Isolation of Mouse Primary Cells

Single cell suspensions from thymus and spleen were generated by mechanical homogenization of indicated tissues in 3 ml of phosphate-buffered saline solution supplemented with 2% bovine serum albumin plus 0.5 mM EDTA (referred to hereafter as cell extraction buffer), washed once by low-speed centrifugation, resuspended in cell extraction buffer, and subjected to 0.17 M NH_4_Cl lysis to eliminate erythrocytes. Bone marrow cells were collected by flushing cell extraction buffer with the aid of syringe into femur and tibia cavities and, subsequently, processed as above.

#### Flow Cytometry

Isolated cells were washed twice in cell extraction buffer, resuspended in standard phosphate-buffered saline solution, and stained with combinations of fluorescein isothiocyanate- (FITC, Cat. No. 553729), allophycocyanin- (APC, Cat. No. 553051), APC-Cy7- (Cat. No. 560181) or V500-labeled (Cat. No. 560783) labeled antibodies to CD4; FITC- (Cat. No. 553031), Pacific blue- (PB, Cat. No. 558106) or phycoerythrin-labeled (PE, Cat. No. 553032) antibodies to CD8; APC- (Cat. No. 558643) or PE-Cy7-labeled (Cat. No. 552880) antibodies to CD25; APC-labeled antibodies to CD24 (Cat. No. 17-0242-82, eBioscience); perinidin chlorophyll-cyanin 5.5-labeled (PerCP-Cy5.5) antibody to B220 (Cat. No. 45-0452-82; eBiosciences); PerCP-Cy5.5-labeled antibody to CD44 (Cat. No. 560570); PE-labeled antibody to TCRβ (Cat. No. 12-5961-82, eBiosciences), PE-Cy7-labeled antibody to human CD5 (Cat. No. 25-0059-41, eBiosciences); PE-labeled antibody to human CD7 (Cat. No. 12-0078-41, eBiosciences), and an APC-labeled antibody to human CD45 (Cat. No. 17-0459-41, eBiosciences).

For intracellular antigen staining, cells were fixed with Cytofix/Cytoperm (Cat. No. 554714, BD Biosciences) for 10 min and stained with PE-labeled antibodies to either TCRβ or ICN1 (mN1A, Cat. No. 552768) for 1 hr at room temperature in phosphate buffered saline solution supplemented with 5% fetal bovine serum and 10% saponin. Unless otherwise stated, the antibodies were obtained from BD Biosciences. Antibody-stained cells were analyzed using a FACSAria III flow cytometer (BD Biosciences) and the FlowJo software.

#### Bioinformatics of Mouse Array Data

R version 3.0.3 was used for the statistical analyses along with Perl for text processing. Signal intensity values were obtained from CEL files after robust multichip average (RMA). Differentially expressed genes were identified using linear models for microarray data (Limma). Adjusted *P*-values for multiple comparisons were calculated applying the Benjamini-Hochberg correction (FDR). Gene Ontology and KEGG pathways enrichment analyses were performed using DAVID. Expression heatmaps were generated using the *heatmap3* package. GSEA were performed with described gene sets using gene set permutations (n = 1000) for the assessment of significance and signal-to-noise metric for ranking genes. To evaluate the *Vav1*^–/–^ T-ALL-associated gene signature fitness across mouse T cell tumors, the enrichment scores for both the upregulated and downregulated signatures were calculated using ssGSEA. The difference between the two normalized enrichment scores yielded the fit score, a measure of the enrichment and depletion of the upregulated and downregulated signatures, respectively. An empirically determined threshold was set to establish the individual samples with moderate (> 0.25) and high (> 0.40) fitness and Bartlett’s, ANOVA and Tukey’s HSD tests were performed to identify the T-ALL subtypes with significant enrichment of the signature. The accession codes for the datasets used are E-MEXP-2737 (ArrayExpress database, EMBL-EBI), GSE28823 (Gene Ommnibus Databse, GEO), GSE19499 (GEO database), GSE12948 (GEO databse) and GSE15907 (GEO database).

#### Determination of mRNA Abundance

Total RNA was extracted from cells using Trizol (Sigma) and analyzed by qRT-PCR using the iScript One-Step RT-PCR kit (BioRad) with SYBR green (BioRad) and the StepOnePlus Real-Time PCR System (Applied BioSystems). Raw qRT-PCR data were analyzed using the StepOne software v2.1 (Applied Biosystems), using the abundance of the endogenous *B2m* and *GAPDH* as internal normalization controls for mouse and human samples, respectively. Primers used were 5’-GGCC AGC TGA TAT AAT GGA GAA AA-3’ (forward) and 5’-TCC ATG ATA GGC TTT GAT GAC TT-3’ (reverse) for mouse *Hes1* cDNA; 5’-TGA AGA ACA TGG CCA AGG GTG AGA-3’ (forward) and 5’-CTG ATG TGT CAT CCG CCT CAT CCT-3’ (reverse) for mouse *Notch3* cDNA; 5’-CGA AAC TCT GGT GCA TAA ACT G-3’ (forward) and 5’-GAA CCG TTC TCC TTA GCT CTC A-3’ (reverse) for mouse *Myc* cDNA; 5’-GGA CAT GCA GAA CAA CAA GC-3’ (forward) and 5’-CAG TCT CAT AGC TGC CCT CA-3’ (reverse) for mouse *Notch1* cDNA (3’); 5’-TGT GCA GCG TGT TAA TGA CT-3’ and 5’-CAG GGC ACC TAC AGA TGA AT-3’ for mouse *Notch1* cDNA (5’); 5’-GCT ATC CAG AAA ACC CCT CAA-3’ (forward) and 5’-CAT GTC TCG ATC CCA GTA GAC GGT-3’ (reverse) for mouse *B2m* cDNA; 5’-TGG TGT CCT TCT GTG TCA GC-3’ (forward) and 5’-CTT GAG GCC GAA CTT CTC AC-3’ (reverse) for human *VAV1* cDNA; 5’-TCA AGG TGC ATC ACA GCT TC-3’ (forward) and 5’-TTC AGT GTG CAC TCC TCG AC-3’ (reverse) for human *VAV2* cDNA; 5’-CTG CAT TTC TGG CTG TTC AA-3’ (forward) and 5’-CTG GGA AGA ACA GCT CTT GG-3’ (reverse) for human *VAV3* cDNA; 5’-TCA ACA CGA CAC CGG ATA AA-3’ (forward) and 5’-CCG CGA GCT ATC TTT CTT CA-3’ (reverse) for human *HES1* cDNA; 5’-GCC GCC TTT GTG CTT CTG TTC-3’ (forward) and 5’-CCG GTG GTC TGT CTG GTC GTC-3’ (reverse) for human *NOTCH1* cDNA; 5’-TTC CAG ATG GCA AAC TCA ATG-3’ (forward) and 5’-TAC ATT CTC TCC TTG CCT TCT TTA-3’ (reverse) for human *CBLB* cDNA; 5’-CCT GGC AGT TAT ATC TTC CGG-3’ (forward) and 5’-TAC CAC GAT GGG TTC AGT ACC-3’ (reverse) for human *CBL* cDNA; 5’-ATG GCC TTC CGT GTC CCC ACT G-3’ (forward) and 5’-TGA GTG TGG CAG GGA CTC CCC A-3’ (reverse) for human *GAPDH* cDNA.

#### Western Blotting

To determine abundance of proteins, primary thymocytes and cancer cell lines were extensively washed with phosphate-buffered saline solution and lysed in 10 mM Tris-HCl (pH 8.0), 150 mM NaCl, 1% Triton X-100, 1 mM Na_3_VO_4_, 10 mM β-glycerophosphate, and a mixture of protease inhibitors (Cømplete, Roche). Cellular extracts were precleared by centrifugation at 14,000 rpm for 10 min at 4°C, denatured by boiling in 2x SDS-PAGE sample buffer, separated electrophoretically, and transferred onto nitrocellulose filters using the iBlot Dry Blotting System (Thermofisher). The same separation and transfer was done in the case of immunoprecipitation experiments (see below). Membranes were blocked in 5% bovine serum albumin (Cat. No. A4503, Sigma) in TBS-T (25 mM Tris-HCl (pH 8.0), 150 mM NaCl, 0.1% Tween-20) for at least 1 hr and then incubated overnight with the appropriate antibodies. Membranes were then washed three times with TBS-T, incubated with the appropriate secondary antibody (1:5,000 dilution, GE Healthcare) for 30 min at room temperature, and washed twice as above. Immunoreacting bands were visualized using a chemoluminescent method (ECL, GE Healthcare). Primary antibodies used included those to the ICN1 fragment (Cat No. 4147, Cell Signaling; 1:1,000 dilution), tubulin α (Cat. No. CP06-100UG, Calbiochem; 1:2,000 dilution), Vav1 DH (homemade, 1:10,000 dilution; used to detect the WT and C-terminally truncated versions of Vav1), Vav1 SH2 (homemade, 1:10,000 dilution; used to detect the Vav1 SH3-SH2-SH3 fragment), HA epitope (Cat. No. MMS-101P, Covance, 1:1,000 dilution), Cbl-b (Cat No. 9498, Cell Signaling; 1:1,000 dilution), GFP (Cat. No. MMS-118P, Covance; 1:2,000 dilution), full length Notch1 (Cat. No. sc-6014, Santa Cruz; 1:1,000 dilution), CD98 (Cat. No. ab108300, Abcam; 1:1,000 dilution), PCNA (Cat. No. ab92552, Abcam; 1:1,000 dilution), GlyRS (Cat. No. sc-98614, Santa Cruz; 1:1,000 dilution), TLX1 (Cat. No. sc-880; Santa Cruz, 1:1,000 dilution), and TLX3 (Cat. No. sc-81990, Santa Cruz, 1:1,000 dilution).

#### Determination of Promoter Activation

To measure stimulation of the Notch1 pathway using luciferase reporter assays, 2 x 10^7^ of exponentially growing Jurkat and HPB-ALL cells were coelectroporated (250 V, 950 μF) with 20 μg of the appropriate expression vectors, the pRL-SV40 vector encoding the *Renilla* luciferase (5 ng) plus either the pCBF-1 (to measure RBPJκ-responsive elements) or pHES1-Luc (to measure *HES1* promoter activity) plasmids (10 μg each). When required, electroporations were supplemented with empty vectors to maintain constant the total amount of transfected DNA among samples. After 48 hr, cells were lysed with Passive Lysis Buffer (5x) and luciferase activities determined using the Dual Luciferase Assay System (Cat No. E1960, Promega). To measure activation of the NFAT pathway, 2 x 10^7^ of exponentially growing Jurkat cells were coelectroporated with 20 μg of the appropriate Vav1-encoding experimental vectors, the pNFAT-luc reporter vector (10 μg) and pRL-SV40 (5 ng). 36 hr posttransfection, cells were either left non-stimulated or stimulated with antibodies to human CD3 (Cat. No. 217570, UCHT1 clone, Calbiochem, 7,5 μg/ml) for 7 hr and then luciferase activities determined as above. In all cases, the values of firefly luciferase activity obtained in each experimental point were normalized taking into account the activity of the *Renilla* luciferase obtained in the same sample. In addition, we analyzed aliquots of the same lysates by Western blot to assess the expression of the ectopically expressed proteins in the appropriate experimental sample. Values are represented in the figures as the n-fold change of the experimental sample relative to the *HES1* promoter, RBPJκ, and NFAT activity shown by control cells (which was given in each case an arbitrary value of 1).

#### ICN1 Ubiquitinylation

Jurkat cells were transiently transfected with pICN1 and pUb-HA plasmids as above and, after 36 hr, incubated with 50 μM MG132 (Cat No. 474790, Calbiochem) for 6 additional hr in standard cell culture conditions. Cells were then lysed in 50 mM HEPES (pH 7.5), 150 mM NaCl, 10% glycerol, 1.5 mM MgCl_2_, 1% Triton X-100, 1 mM EGTA, 10 mM Na_3_VO_4_, 100 mM NaF, Cømplete, and 10 μM MG132 at 4°C. Upon elimination of cell debri by centrifugation, cellular extracts were incubated for 2 hr at 4°C with anti-HA (1:100 dilution) followed by 1 hr of incubation with Gammabind G-Sepharose beads (GE Healthcare) at 4°C. After washes, immunocomplexes were subjected to Western blot analyses as indicated above.

#### In Vivo γ-Secretase Activity

3 × 10^7^ exponentially growing Jurkat cells were collected in ice-cold phosphate-buffered saline solution and pelleted at 5,000 rpm for 5 min. The pellet was homogenized in 500 μl of γ-secretase assay buffer (20 mM HEPES (pH 7.5), 150 mM KCl, 2 mM EGTA, 1 mM Na_3_VO_4_, 10 mM β-glycerophosphate and Cømplete) and passed through a 27 G needle five times using a 1 ml syringe. The resulting homogenate was cleared at 45,000 rpm for 1 hr at 4°C. The pellet from this step was resuspended in 500 μl of γ-secretase assay buffer and passed through a 27 G needle five times on ice. The suspension was cleared at 45,000 rpm for 1 hr at 4°C and, upon elimination of the resulting supernatant, the pellet was resuspended in 75 μl of γ-secretase assay buffer supplemented with 1% CHAPSO and subjected to rotation for 2 hr at 4°C. The solubilized fraction from that step was centrifuged at 45,000 rpm for 1 hr at 4°C and the resulting supernatant considered the membrane-enriched fraction to be used in the γ-secretase activity determination. To this end, we monitored changes in the fluorescence emission of a fluorogenic peptide containing the amyloid precursor protein γ-secretase cleavage site (Cat. No. 565764, Calbiochem) upon an incubation with either cellular membranes or bovine serum albumin (negative control) for 16 hr at 37°C in a 5% CO_2_ atmosphere (Farmery et al., 2003). To demonstrate bona fide γ-secretase activity, we incubated in parallel aliquots from the same samples with 200 nM Compound E. In all cases, fluorescence changes (excitation max.: ≈355 nm; emission max.: ≈440 nm) in the incubation mixture were measured using a microplate reader (Ultraevolution, Tecan).

#### Immunoprecipitation Experiments

In the case of endogenous proteins, 5 × 10^7^ exponentially growing Jurkat cells were lysed in 10 mM Tris-HCl (pH 8.0), 150 mM NaCl, 1% Triton X-100, 1 mM Na_3_VO_4_, 10 mM β-glycerophosphate and Cømplete. Upon elimination of cell debri by centrifugation, cellular extracts were incubated for 2 hr at 4°C with primary antibodies. In some cases, cells were treated with 5 mM ivermectin (Cat. No. I8898, Sigma) and 50 μM MG132 for 4 hr prior to the lysis step. Upon elimination of cell debri by centrifugation, cellular extracts were incubated for 2 hr at 4°C with antibodies to ICN1 (Cat No. ab52301, Abcam; 1:100 dilution). In the case of ectopically expressed proteins, 2 × 10^6^ of exponentially growing Jurkat cells were transfected with 20 μg of the appropriate mammalian expression vector (diluted in 2 ml of R buffer; Cat No. MPK10096, Life Technologies) using two 20-msec electroporation cycles at 1.7 mV in the Neon system (Life Technologies). Electroporated cells were then maintained in standard culture media for 36 hr and lysed in 10 mM Tris-HCl (pH 8.0), 150 mM NaCl, 1% Triton X-100, 1 mM Na_3_VO_4_, 10 mM β-glycerophosphate and Cømplete. Upon elimination of cell debri, cellular extracts were incubated for 2 hr at 4°C with primary antibodies. Those included antibodies to HA (Cat. No. MMS-101P, Covance, 1:100 dilution), Cbl-b (Cat. No. sc-1435, Santa Cruz, 1:200 dilution), and ICN1 (Cat No. ab52301, Abcam; 1:100 dilution). In all cases, immunocomplexes were collected with Gammabind G-Sepharose beads (GE Healthcare), washed three times in the buffer used for cell lysis, resuspended in SDS-PAGE buffer, boiled for 5 min, and subjected to immunoblot analysis as indicated above.

#### shRNA-Mediated Transcript Knockdowns

To knockdown *VAV1*, the indicated T-ALL cell lines were infected with lentiviruses encoding either scrambled (TR1.5-pLKO-1-puro, Sigma) or *VAV1*-directed shRNAs (TRCN0000039858 [referred to in the figures as sh1], TRCN0000039859 [referred to in the figures as sh2], TRCN0000039860 [referred to in the figures as sh3]; Sigma). To knockdown *CBLB*, Jurkat cells were infected with lentiviruses encoding *CBLB*-specific shRNAs (TRCN000007750 [referred to in the figures as sh1], TRCN000007751 [referred to in the figures as sh2], TRCN000007752 [referred to in the figures as sh3], TRCN0000011198 [referred to in the figures as sh4]; Sigma). To inactivate TLX1, ALL-SIL cells were transduced with lentiviral particles encoding a *TLX1*-directed shRNA (TRCN0000014995; Sigma). To knockdown TLX3, HBP-ALL cells were infected with lentiviruses encoding a *TLX3*-specific shRNA (TRCN0000018030; Sigma). In all cases, cells were subjected to either long-term (15 days; Jurkat, CEM, and Molt4) or short-term (5 days; ALL-SIL, HPB-ALL) puromycin selection. Proper transcript knockdown was assessed using immunobloting and/or qRT-PCR.

#### Subcellular Fractionation

Jurkat cells (3 × 10^7^) were resuspended in 1 ml of hypotonic lysis buffer (10 mM Tris-HCl (pH 8.0), 150 mM NaCl, 1 mM Na_3_VO_4_, 10 mM β-glycerophosphate and Cømplete) and passed through a 25 G needle 10 times using a 1 ml syringe. Cells were then centrifuged at 14,000 rpm for 10 min at 4°C. The pellet (P1) from this step was resuspended in 500 μl of hypotonic lysis buffer supplemented with 1% Triton X-100 and subjected to centrifugation at 3,000 rpm for 10 min at 4°C to eventually collect the resulting supernatant and pellet fractions that were considered, respectively, as the nuclear and insoluble compartments. The supernatant from the first centrifugation step (S1) was diluted to a final volume of 5 ml with the hypotonic lysis buffer and subjected to high-speed (60,000 rpm) centrifugation for 1 hr at 4°C using polycarbonate centrifuge tubes (Cat. No. 349622, Beckman Coulter). The supernatant and pellet fractions from this step were considered as the cytosolic and membrane compartment, respectively. All fractions were resuspended in SDS-PAGE buffer, boiled for 5 min, and subjected to immunoblot analysis as indicated above. In the case of experiments to check the subcellular distribution of ICN1 ubiquitinylation, Jurkat cells were transiently transfected with the pICN1 and pUb-HA expression plasmids (see section above) and, 48 hr later, subjected to the subcellular fractionation procedure indicated for nontransfected cells.

#### Bioinformatics of Human T-ALL Microarray Data

Expression heatmaps were generated as above, using GEO datasets GSE62156 (dataset 1), GSE28703 (dataset 2) and GSE26713 (dataset 3). In overall, these arrays include a total of 240 patients, either adult (dataset 1 and 2; n = 64 and 52, respectively) or pediatric (dataset 3, n = 124). TLX status in those samples was determined according to genome chacterization (dataset 1 and 3). However, since these data were missing in dataset 2, we defined as “TLX^+^” in this case the samples that showed high abundance of either *TLX1* or *TLX3* trancripts according to expression heatmap analyses.

To carry out ssGSEA, we first generated a shared gene signature composed of genes found deregulated in tumor cells from both *Vav1*^–/–^ (this work) and *Zfp36l1*^–/–^;*Zfp36l2*^–/–^ (ArrayExpress E-MEXP-2737) knockout mice. To this end, we performed a GSEA on the *Vav1*^–/–^ expression dataset using as gene set the differentially expressed genes (absolute fold change > 2) in *Zfp36l1*^–/–^;*Zfp36l2*^–/–^ knockout mouse-derived microarray-derived samples. The analysis of the up- and downregulated gene sets yielded a series of genes belonging to the leading edge (core enrichment), which was referred as the “shared *Vav1*^–/–^*/Zfp36l1*^–/–^;*Zfp36l2*^–/–^ gene signature” (Table S3). This signature was used to test possible hits with array data from T-ALL dataset 1 (GEO GSE62156) using both ssGSEA and GSEA (Figures S7C–S7E). Subsequently, it was further refined to eliminate deregulated genes that could be associated with just a normal undifferentiated state of T cells. To generate this “tumor-specific” signature, we selected genes that, according to GSEA performed with T-ALL dataset 1 and a T-cell development microarray dataset (GEO GSE15907), were not associated with the undifferentiated state of “healthy” T lymphocytes (Figure S7F and Table S3). The presence of this “tumor-specific” *Vav1*^–/–^/*Zfp36l1*^–/–^;*Zfp36l2*^–/–^ gene signature in samples from T-ALL datasets 1, 2 and 3 was determined using ssGSEA as described above. Expression heatmaps were generated as indicated above for mouse array analyses.

Expression correlation matrices were calculated for the indicated mRNA pairs using the *corrplot* package (http://CRAN.R-project.org/package=corrplot) and the datasets 1, 2 and 2 indicated above. Correlations were considered as statistically significant when the Pearson correlation coefficient corresponded to a p value below 0.05. Depending upon the total number of samples in each dataset, this significance was achieved when the absolute value for the Pearson correlation coefficient was above 0.39 (dataset 1), 0.33 (dataset 2) and 0.28 (dataset 3).

#### Transcriptional Factor Binding to Regulatory Gene Sequences

In the case of Chip-seq analyses, raw data mapping and peak calling were performed with Bowtie and MACS1.4, selecting peaks with p value ≤ 10^−5^. hg18 coordinates were converted to the hg19 assembly using the LiftOver utility of the UCSC Genome Browser Tools. Wig files were converted to bed format using the wig2bed utility of the BEDOPS Suite. Visualization of data was performed in R using the Gviz package. The accession codes for the datasets used are GSE62144 (for TLX1) and GSE51800 (for Ets1 and Runx1). In the case of ChIP-chip analyses with TLX1 and TLX3, we used the dataset reported before (Della Gatta et al., 2012). ChIP-chip Significance Analysis was used, applying a significance cutoff of p ≤ 10^–9^ to select statistically significant peaks.

#### Lentiviral-Mediated Expression of EGFPs in T-ALL Cells

For EGFP-Vav1^WT^ overexpression, T-ALL cells were infected with lentiviral particles encoding either EGFP or the indicated EGFP-Vav1 fusions by spinoculation of viral supernatants produced in HEK293T cells at 1,800 rpm for 90 min without brake at room temperature. Proper protein expression was assessed using flow cytometry.

#### Determination of Cell Proliferation

HPB-ALL, Jurkat, Molt4, and Loucy cells were transfected with the indicated vectors using the Neon system. ALL-SIL cells were infected with either EGFP- or EGFP-Vav1^WT^-encoding lentiviruses. At the indicated time points, proliferation was measured in all cases using the 3-(4,5-dimethylthiazol-2-yl) 2,5-diphenyltetrazolium bromide (MTT) method. To this end, the culture medium of each well was replaced by 100 μl of the MTT solution (0.5 mg/ml) made in phosphate-buffered saline solution. After 2  hr at 37 °C in a 5% CO_2_ atmosphere, 100  μl of DMSO were added per well to dissolve the formazan crystals formed and the absorbance at 570 nm measured 15 min later using the Ultraevolution reader.

#### Determination of Apoptotic Rates

Cells were harvested either 48-96 (in the case of HPB-ALL, ALL-SIL, Loucy and Jurkat cells) or 48 (in the case of patient-derived T-ALL cancer cells) hr after the transfection or viral transduction step respectively, stained using the Annexin V–fluorescein propidium isothiocyanate detection kit (Immunostep), and apoptosis determined in the population of GFP-gated cells using flow cytometry.

### Quantification and Statistical Analysis

#### Statistics

Tukey’s HSD tests were used to identify those groups showing differential enrichment of the indicated signatures. Student’s t and Mann-Whitney tests were used to analyze results from wet lab experiments as indicated in the figure legends. Statistical analyses were carried out using the R (in the case of Tukey’s HSD tests) and GraphPad Prism software (version 6.0; in the case of both Student’s t and Mann-Whitney tests). The number of biological replicates (n) and significance for each experiment can be found in the figure legend of the experiment as well as the results section of this document.

### Data and Software Availability

#### Data Resource

Microarray data reported in this paper has been deposited in the GEO database (https://www.ncbi.nlm.nih.gov/geo/) under the accession number GSE80490.

## Author Contributions

J.R.-V. participated in all experimental work, analyzed data, and contributed to artwork design and manuscript writing. L.F.L.-M. carried out bioinformatics analyses. M.M.-M. initiated the study. I.F.-P. helped with the generation and analysis of shRNA-expressing T-ALL cells. A.A. carried out animal-related procedures. M.C. and M.L.T. provided human T-ALL samples. L.E. and A.B. generated reagents and analyzed data. X.R.B. conceived the work, analyzed data, wrote the manuscript, and performed the final editing of figures.

## Supplemental Information

Document S1. Figures S1–S8 and Tables S2 and S4

Table S1. List of Genes Differentially Regulated in *Vav1*^−/−^ T Cell Tumors, Related to Figure 2

Table S3. List of the Genes Belonging to the “Shared” and “Tumor-Specific” *Vav1*^*−/−*^*/Zfp36l1*^*−/−*^;*Zfp36l2*^*−/−*^ Gene Signatures, Related to Figure 7

Document S2. Article plus Supplemental Information
