# Supplementary material for: A Paradoxical Tumor-Suppressor Role for the Rac1 Exchange Factor Vav1 in T Cell Acute Lymphoblastic Leukemia
Source: Cancer Cell. 2017 Nov 13;32(5):608–623.e9. doi: 10.1016/j.ccell.2017.10.004 (PMC5691892; doi:10.1016/j.ccell.2017.10.004)
Supplement: Document S1. Figures S1–S8 and Tables S2 and S4 [file mmc1.pdf]

Cancer Cell, Volume *32*

Supplemental Information

A Paradoxical Tumor-Suppressor Role for the Rac1 Exchange Factor Vav1 in T Cell Acute Lymphoblastic Leukemia

Javier Robles-Valero, L. Francisco Lorenzo-Martín, Mauricio Menacho-Márquez, Isabel Fernández-Pisonero, Antonio Abad, Mireia Camós, María L. Toribio, Lluis Espinosa, Anna Bigas, and Xosé R. Bustelo

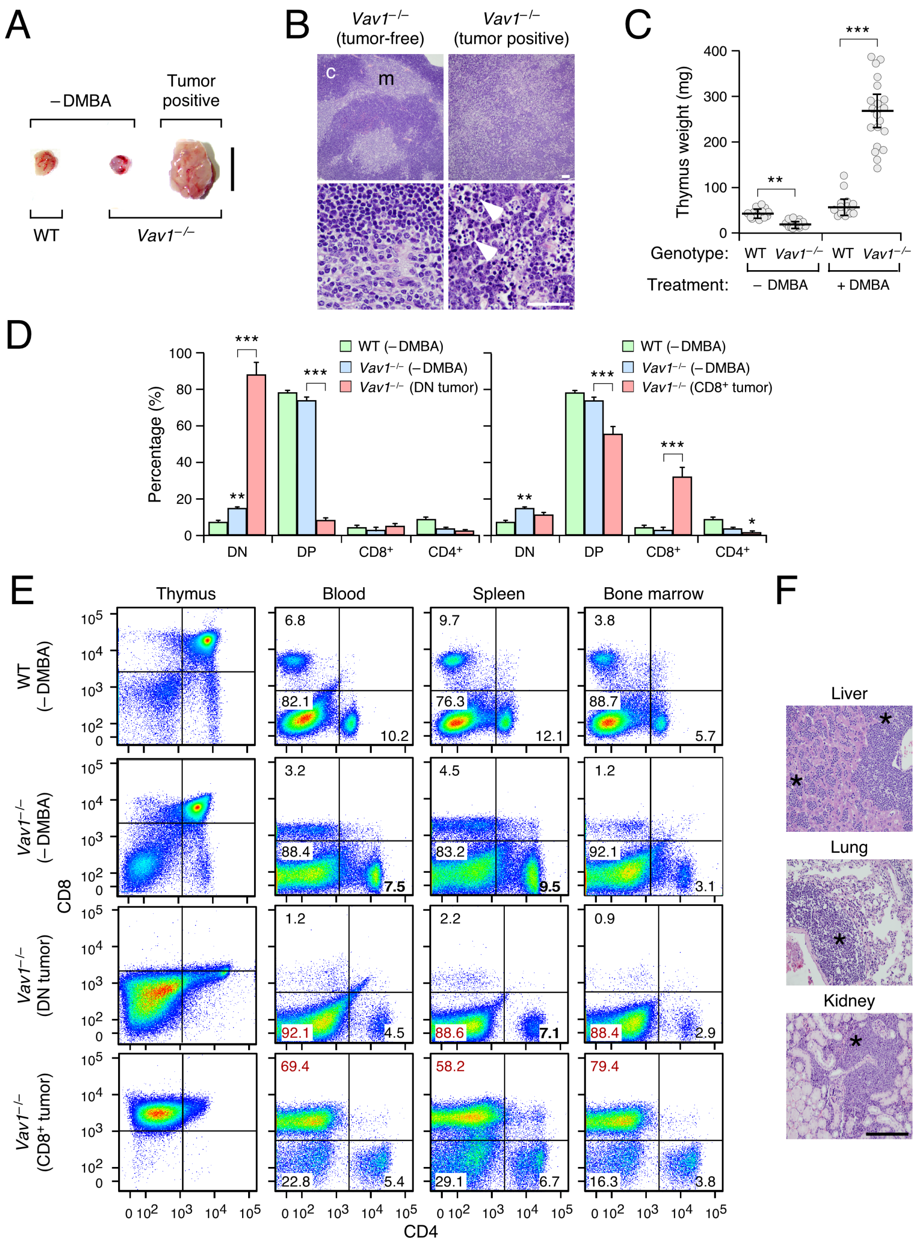

Figure S1, related to Figure 1. Characterization of T cell tumors developed in DMBA-treated *Vav1*$^{-/-}$ mice. **(A)** Examples of thymi isolated from indicated mice. Vertical scale bar on the right, 1 cm. **(B)** Hematoxylin-eosin stained sections of thymi from a healthy *Vav1*$^{-/-}$ (left panel) and a tumor–bearing *Vav1*$^{-/-}$ (right panel) mouse, respectively. Arrows indicate the presence of macrophages containing apoptotic cells. Scale bars, 10 (top panels) and 100 (bottom panels) µm. c, cortex; m, medulla. **(C)** Weight of thymi from mice of indicated genotypes and experimental conditions. Each point represents the measurement of an individual mouse (n = 15 mice each for WT –DMBA, *Vav1*$^{-/-}$ –DMBA and WT +DMBA, 20 mice for *Vav1*$^{-/-}$ +DMBA). **(D)** Percentage of indicated thymocyte populations (bottom) in control and tumor-bearing mice determined by flow cytometry. Data from DN (left) and CD8^{+} (right) tumor-bearing animals is included. In panels C and D, data represents the mean ± SEM. Statistical values are given relative to either untreated WT controls or indicated experimental pairs (in brackets). *, $p \leq 0.05$; **, $p \leq 0.01$; ***, $p \leq 0.001$ (Mann-Whitney and Student's t tests for panel C and D, respectively). **(E)** Examples of the distribution of CD4 and

CD8 surface markers in samples from indicated mice (left), and tissues (top). Numbers in each quadrant indicate the relative percentage (in %) of each cell population. Red color indicates abnormally expanded T cell subpopulations. **(F)** Representative example of hematoxylin-eosin stained liver, lung and kidney sections from a $CD8^+$ tumor–bearing *Vav1*$^{-/-}$ mouse. Asterisks indicate the localization of metastatic cells. Scale bar, 10 μm.

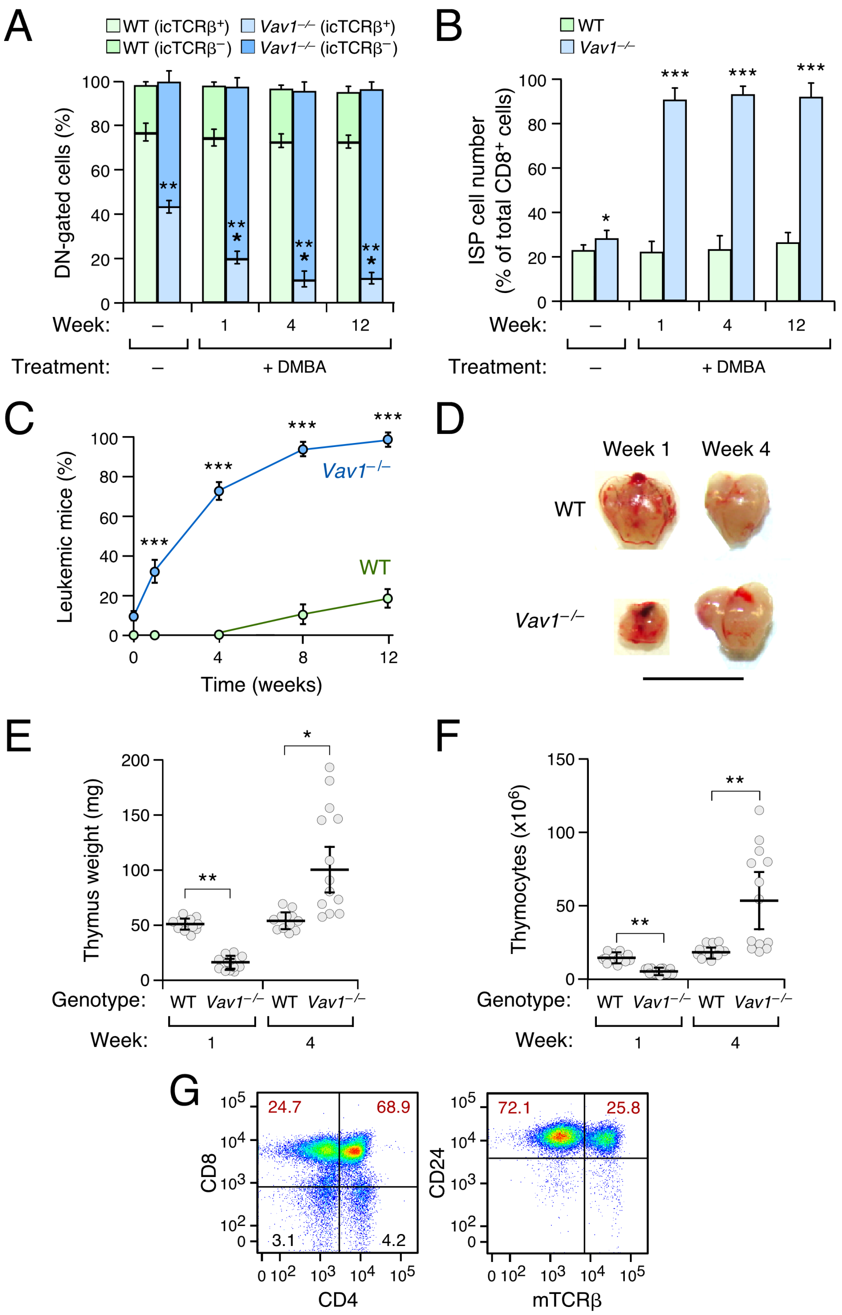

Figure S2, related to Figure 1. Characterization of *Vav1*$^{-/-}$ mice during early times after DMBA treatments. (A,B) Flow cytometry-determined percentages of DN-gated TCRβ^{+} (A) and ISP (B) cells present in indicated mice (top) and post-DMBA treatment times (bottom) (n = 10 animals per genotype in the first three experimental conditions, 15 animals per genotype in the 12 week+DMBA experimental condition). **(C)** Percentage of DMBA-treated mice of indicated genotypes with detectable expansion of DN or CD8^{+} cell populations at indicated experimental time-points. (n = 15 animals per genotype). **(D)** Representative examples of thymi from mice of the indicated genotypes at the indicated DMBA posttreatment times. Scale bar, 1 cm. **(E,F)** Thymus weight (E) and thymocyte numbers (F) in indicated mice and DMBA posttreatment periods (bottom). Each point represents the measurement of an individual mouse (n = 13 per experimental condition in each case). **(G)** Flow cytometry of a CD8^{+} tumor-bearing WT mouse upon staining with

antibodies to CD4 and CD8 (left) as well as $CD8^+$-gated thymocytes from the same tumor upon staining with antibodies to CD24 and mTCRβ (right). Numbers in each quadrant indicate the relative percentage (%) of each cell population. In panels A to C, E and F, bars represent the mean ± SEM. Statistical values are given relative to either untreated WT controls or indicated experimental pairs (in brackets). *, $p \leq 0.05$; **, $p \leq 0.01$; ***, $p \leq 0.001$ (calculated using Student's t test in the case of panels A to C and Mann-Whitney test in the case of panels E and F).

Table S1, related to Figure 2, is provided as an Excel file.

Table S2, related to Figure 2. Mutations in *Notch1*, *Fbxw7* and *Pten* found in 20 independent tumors from DMBA-treated *Vav1*$^{-/-}$ mice

Gene	Tumor sample	Nucleotide site (mutation)	Homozygosis/ Heterozygosis	Targeted exon	Mutation in protein	Detected in human tumors*	Tumor type*
Notch1	#3	7314 (G>T)**	Homozygous	34	Ala2438Ser (PEST region)	No	–
	#15	4788 (A>T)***	Homozygous	26	Ser1596Cys (HD domain)	Yes (Ser1597Thr, Ser1597Ile)	Hematopoietic
	#17	4869 (A>T)***	Homozygous	26	Glu1623Lys (HD domain)	Yes (Glu1623Lys, Glu1623*)	Skin, upper aerodigestive tract
	#1-2, #4-14, #16, #18-20	None	–	–	None	–	–
Fbxw7	#12	1110 (G>A)****	Heterozygous	8	Glu370Lys (1st WD40)	No	–
	#1-11, #13-20	None	–	–	None	–	–
Pten	#4	Deletion	Homozygous	9	Truncation	Not investigated	Not investigated
	#5	Deletion	Homozygous	8 & 9	Truncation	Not investigated	Not investigated
	#1-3, #6-20	None	–	–	None	Not investigated	Not investigated

* According to data currently present in the COSMIC and cBioPortal databases.
** Secondary mutations arising in DMBA-induced tumors due to either increased genomic instability or oxidative stress.
*** Transversion mutation typically induced by DMBA.
**** Mutation usually triggered by alkylating agents and defective DNA repair.

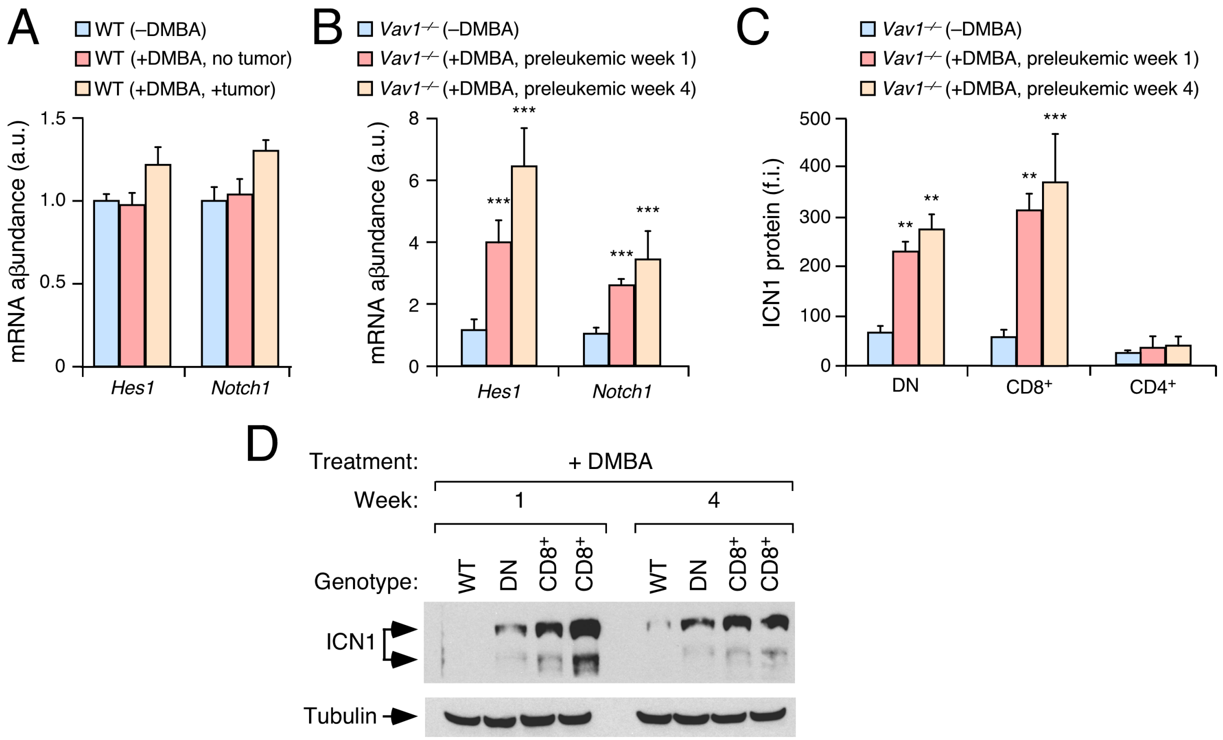

Figure S3, related to Figure 2. ***Vav1*$^{-/-}$ T-ALL shows constitutive activation of the Notch1 pathway**. **(A,B)** qRT-PCR determination of Notch1 downstream targets (bottom) in indicated experimental samples (insets). Values are given relative to the abundance of each transcript found in *Vav1*$^{-/-}$ controls (non DMBA treated) (n = 15 animals per class analyzed). **(C)** Flow cytometry determination of ICN1 levels in thymocyte populations (bottom) from indicated mice and experimental conditions (inset). In A to C, data represents the mean ± SEM. Statistical values are given relative to untreated *Vav1*$^{-/-}$ mouse controls. **, $p \leq 0.01$; ***, $p \leq 0.001$ (Student's t test). **(D)** Abundance of ICN1 (top) and tubulin α (loading control, bottom) in thymocyte lysates from indicated mice and experimental conditions (top).

A

Figure	Protein	Lane	Mean value (a.u.)	p value	n
4A	Vav1	1	1.00±0.18	-	4
		2	0.18±0.12	0.0047 **	4
		3	1.97±0.29	0.043 *	4
4A	ICN1	1	1.00±0.27	-	4
		2	2.97±0.55	0.009 **	4
		3	0.75±0.19	0.456	4
4G	Ub-ICN1	1	1.00±0.33	-	3
		2	0.18±0.12	0.0018 **	3
		3	0.91±0.08	0.716	3
4I (upper panel)	Vav1	1	1.00±0.09	-	3
		2	0.13±0.05	0.0072 **	3
		3	0.08±0.02	0.0001 ***	3
4I (upper panel)	ICN1	1	1.00±0.33	-	3
		2	4.88±0.27	0.0007 ***	3
		3	4.11±0.34	0.0009 ***	3
4I (bottom panel)	Vav1	1	1.00±0.16	-	3
		2	0.15±0.03	0.0044 **	3
		3	0.11±0.06	0.0008 ***	3
4I (bottom panel)	ICN1	1	1.00±0.26	-	3
		2	3.27±0.46	0.0005 ***	3
		3	3.10±0.22	0.0007 ***	3
S4B	Vav1	1	1.00±0.26	-	3
		2	0.09±0.05	0.0002 ***	3
		3	0.42±0.13	0.0081 **	3
		4	0.11±0.07	0.0009 ***	3
S4B	ICN1	1	1.00±0.19	-	3
		2	4.12±0.66	0.0002 ***	3
		3	2.08±0.25	0.0211 *	3
		4	3.88±0.45	0.0005 ***	3
S4B	Cbl-b	1	1.00±0.12	-	3
		2	1.21±0.17	0.015	3
		3	1.01±0.02	0.716	3
		4	1.02±0.06	0.717	3

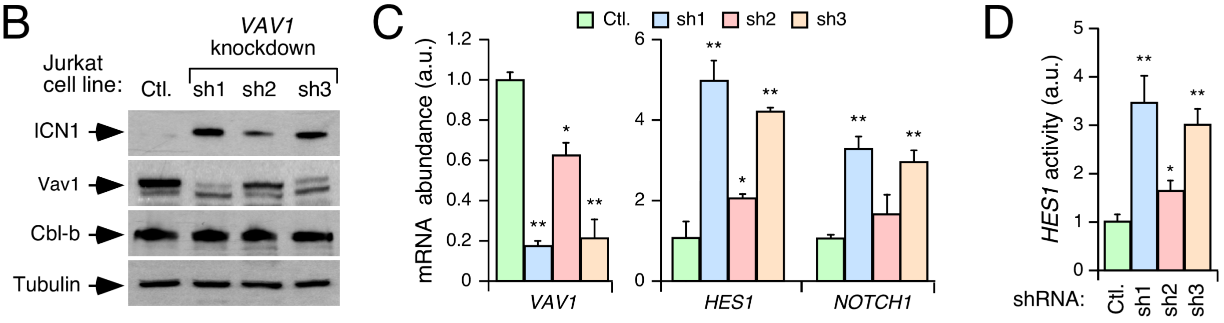

Figure S4, related to Figure 4. Vav1 regulates ICN1 degradation. **(A)** Statistics of Western blot data shown in indicated figures of this work. **(B)** Abundance of endogenous ICN1 (top panel), Vav1 (second panel from top), Cbl-b (third panel from top), and tubulin α (loading control, bottom panel) in TCLs from Jurkat cells stably expressing a control (Ctl.) and three independent (sh1, sh2, sh3) *VAV1* shRNAs (top). **(C)** Abundance of indicated transcripts in the Jurkat cell pools used in panel A (n = 3). **(D)** *HES1* promoter activity in Jurkat cells expressing the indicated shRNAs (bottom) (n = 3). In panels A, C and D, data represent the mean ± SEM. *, p ≤ 0.05; **, p ≤ 0.01; ***, p ≤ 0.001 (Student's t tests).

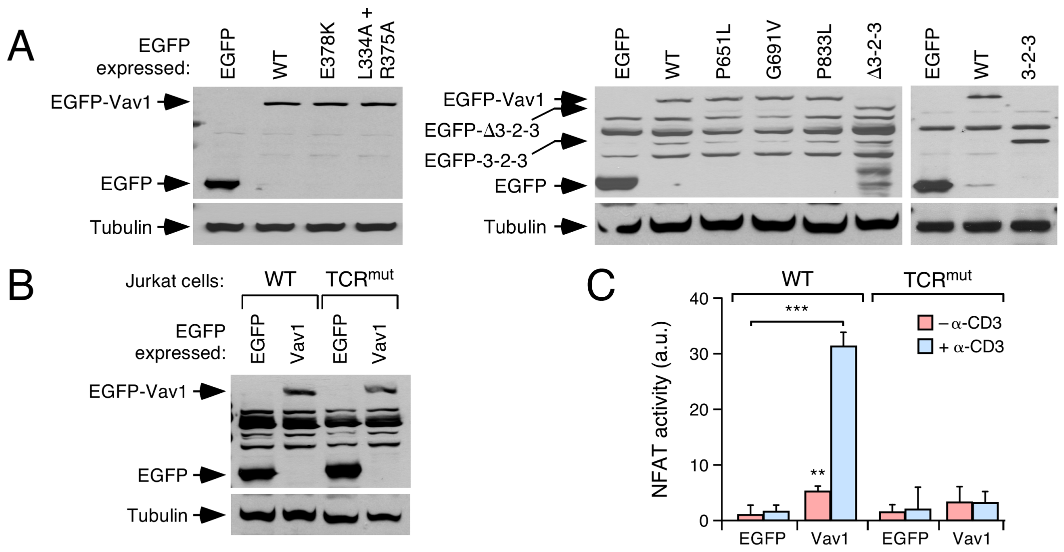

D

Figure	Protein	Lane	Mean value (a.u.)	p value	n
5D	Cbl-b	1	1.00±0.07	-	3
		2	0.16±0.09	0.0072 **	3
		3	0.08±0.05	0.0038 **	3
		4	1.08±0.11	0.891	3
		5	0.93±0.17	0.292	3
5D	ICN1	1	1.00±0.13	-	3
		2	4.12±0.35	0.0008 ***	3
		3	3.97±0.47	0.0019 **	3
		4	1.12±0.09	0.223	3
		5	1.08±0.19	0.452	3
5H	Ub-ICN1	1	1.00±0.21	-	3
		2	0.24±0.13	0.0074 **	3
		3	0.16±0.09	0.0021 **	3

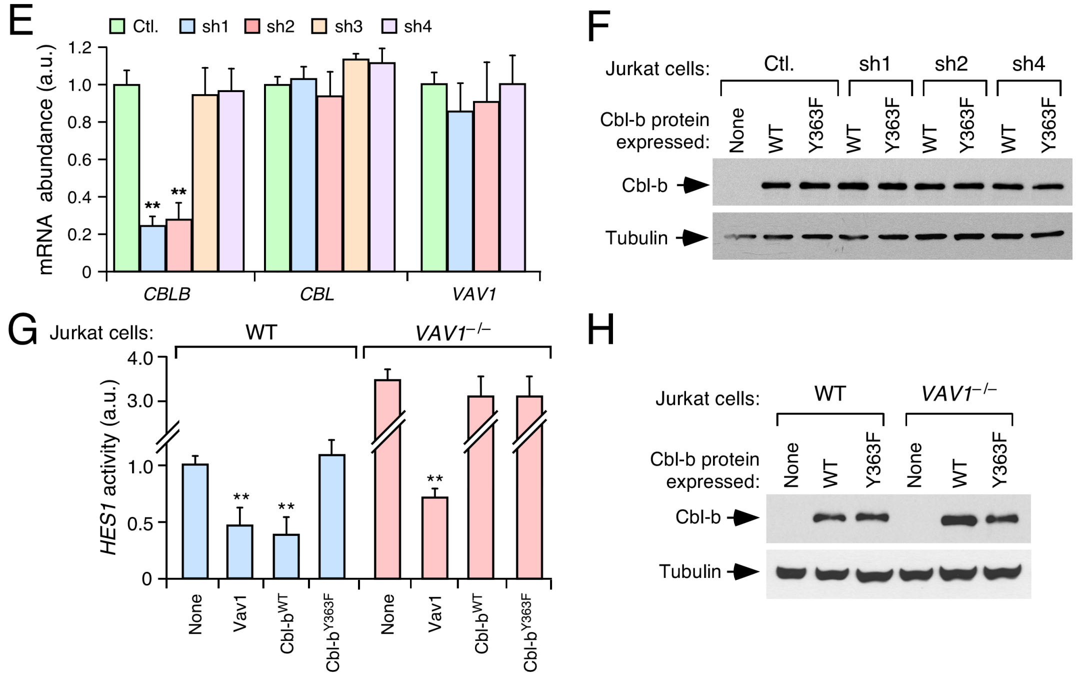

Figure S5, related to Figure 5. Vav1 modulates ICN1 in a Cbl-b-dependent manner. **(A,B)** Immunoblots showing the abundance of the ectopically expressed proteins (top) and endogenous tubulin α (loading control, bottom) in TCLs obtained from the experiments shown in **Figures 5B** (A) and **5C** (B), respectively. Data are from a single representative experiment in each case. EGFPs were detected using antibodies to GFP. **(C)** NFAT reporter

activity in nonstimulated (–α-CD3) and stimulated (+α-CD3) WT and TCR^{mut} Jurkat cells expressing the indicated EGFPs (bottom). Values are given relative to WT Jurkat cells (n = 3). Expression controls are shown in panel B. **(D)** Statistics of Western blot data shown in indicated figures. **(E)** Abundance of *CBL* family and *VAV1* transcripts (bottom) in indicated shRNA-bearing Jurkat cells (insets). Cells used have been defined in **Figure 5D**. Values are given relative to expression in WT Jurkat cells (n = 3). Please, note that the abundance of the transcript for the Cbl-b-like Cbl does not change in *CBLB* knockdown cells. **(F)** Abundance of ectopically expressed Cbl-b (top) and endogenous tubulin α (bottom) in TCLs obtained from one representative experiment used to generate the data shown in **Figure 5G**. **(G)** *HES1* promoter activity of the indicated Jurkat cells (top) expressing either an empty vector or plasmids encoding the indicated Vav1 and Cbl-b proteins (bottom). Values are given relative to WT Jurkat cells (n = 3). **(H)** Abundance of ectopically expressed Cbl-b (top) and endogenous tubulin α (bottom) in TCLs obtained from one representative experiment used to generate the data shown in panel F. In panels C to E and G, data represent the mean ± SEM. **, $p \leq 0.01$; ***, $p \leq 0.001$ using Mann-Whitney (panel C) and Student's t (panels D, E and G) tests.

A

Figure	Protein	Lane	Mean value (a.u.)	p value	n
6A	Vav1	1	0.0	-	3
		2	0.0	-	3
		3	1.00±0.05	-	3
		4	0.22±0.03	0.0033 **	3
		5	1.02±0.02	0.899	3
		6	0.0	0.0001 ***	3
		7	0.0	0.0001 ***	3
		8	0.81±0.09	0.098	3
6C	Vav1	1	1.00±0.07	-	3
		2	0.16±0.09	0.0072 **	3
		3	0.08±0.05	0.0038 **	3
		4	1.08±0.11	0.891	3
		5	0.93±0.17	0.292	3
6D	Vav1	1	0.0	-	3
		2	0.0	-	3
		3	1.00±0.07	-	3
		4	0.0	0.0001 **	3
		5	0.96±0.10	0.733	3
		6	0.0	0.0001 ***	3
		7	0.0	0.0001 ***	3
		8	0.91±0.03	0.455	3
6F	Vav1	1	0.0	-	3
		2	1.00±0.04	-	3
		3	1.02±0.08	0.167	3
		4	0.0	0.0001 ***	3
		5	0.0	0.0001 ***	3
		6	0.0	0.0001 ***	3
		7	0.0	0.0001 ***	3
6G	Cbl-b	1	1.00±0.09	-	3
		2	0.09±0.02	0.0001 ***	3
		3	1.13±0.07	0.345	3
		4	1.19±0.11	0.234	3
		5	0.12±0.06	0.0001 **	3
		6	0.67±0.17	0.0323 *	3
6H	Vav1	1	0.12±0.01	-	3
		2	1.00±0.12	-	3
		3	0.09±0.04	-	3
		4	1.17±0.12	0.127	3

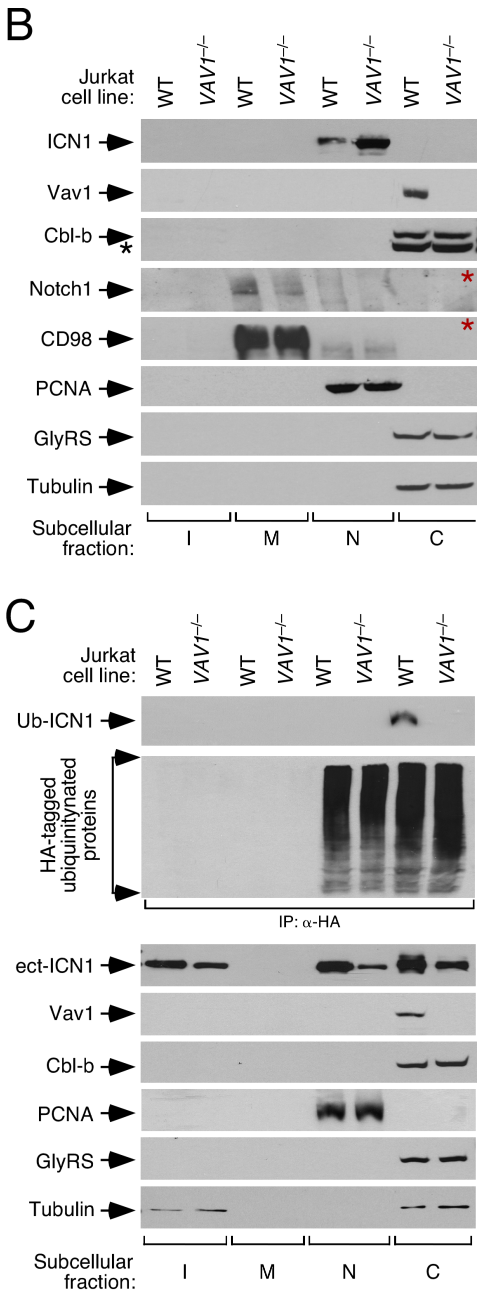

Figure S6, related to Figure 6. Vav1 nucleates a cytoplasmic complex containing Cbl-b and ICN1. **(A)** Statistics of Western blot data shown in **Figure 6**. Reference lanes for statistics are shaded. *, p ≤ 0.05; **, p ≤ 0.01; ***, p ≤ 0.001 (Student's t tests). **(B)** Immunoblots of insoluble (I), membrane (M), nuclear (N) and cytosolic (C) fractions of indicated Jurkat cell lines (top) showing the localization of selected experimental proteins and subcellular localization markers (left). The black asterisk in the Cbl-b panel labels the GlyRS (Glycyl-tRNA synthetase) protein band from the previous immunoblot performed in the same filter. Red asteriscs label panels that have been generated using electrophoresed lysates transferred to an independent nitrocellulose filter. **(C)** Detection of ubiquitinylated, ectopically expressed ICN1 (top panel) and endogenous proteins (second panel from top) in indicated Jurkat cells (top) and subcellular fractions (bottom). As control, aliquots of the lysates used in the above immunoprecipitations were analyzed by immunoblot to detect the amount of ectopically expressed ICN1 (third panel from top) and the indicated subcellular fractionation controls (fourth to eight panels from top). In B and C, similar data were obtained in an additional independent experiment. Quality of the fractionation procedure was demonstrated using antibodies to membrane (CD98), cytoplasmic (tubulin, GlyRS), and nuclear (PCNA) markers. All blots were generated using

antibodies to the indicated proteins. The only exception is the detection of total ubiquitinylated proteins (C, second panel from top), which was carried out using antibodies to the HA epitope.

Table S3, related to Figure 7, is provided as an Excel file.

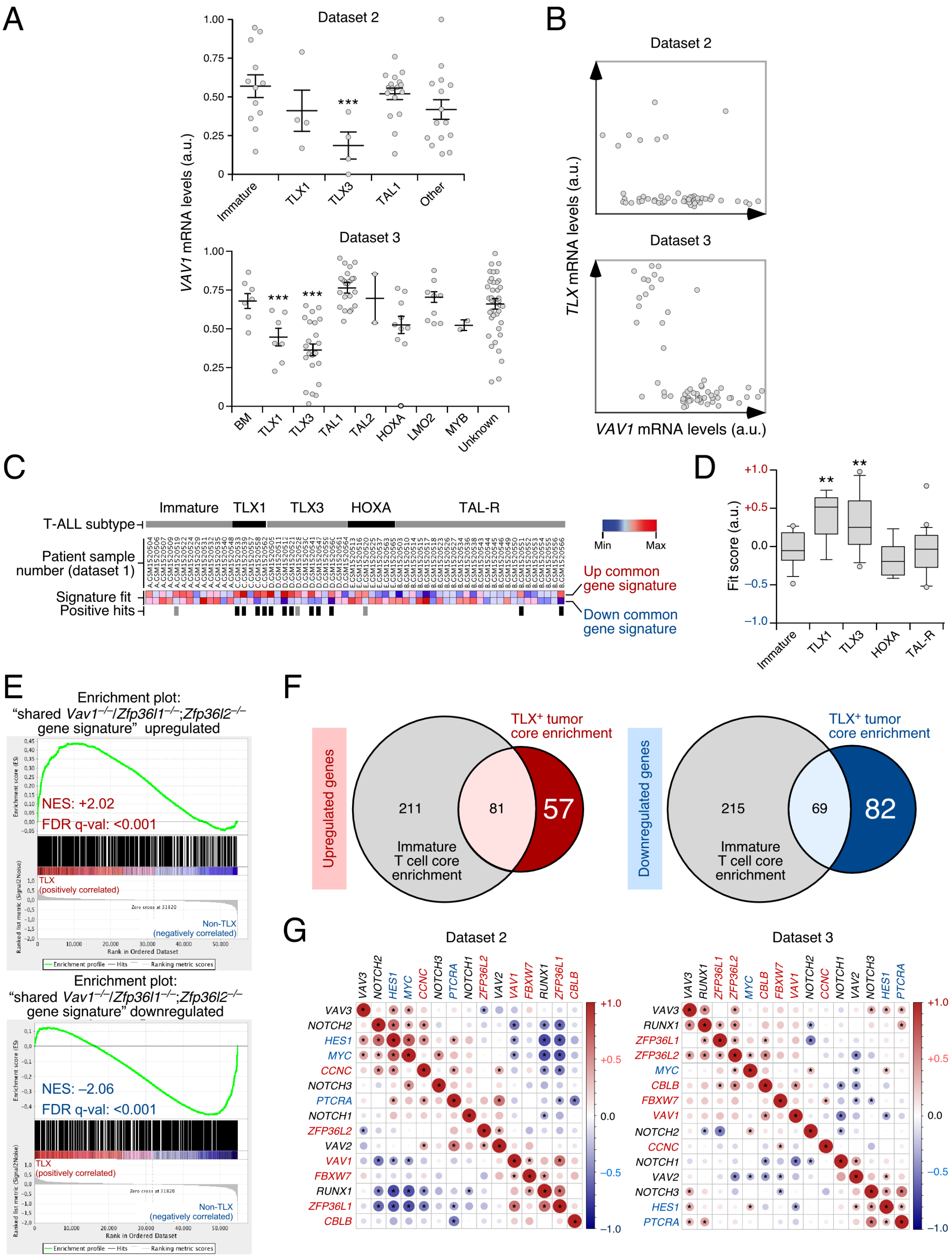
A
Dataset 2
VAV1 mRNA levels (a.u.)
1.00
0.75
0.50
0.25
0
***
Immature
TLX1
TLX3
TAL1
Other
Dataset 3
***
***
BM
TLX1
TLX3
TAL1
TAL2
HOXA
LMO2
MYB
Unknown
B
Dataset 2
Dataset 3
TLX mRNA levels (a.u.)
VAV1 mRNA levels (a.u.)
C
T-ALL subtype
Immature
TLX1
TLX3
HOXA
TAL-R
Patient sample number (dataset 1)
Signature fit
Positive hits
Min
Max
Up common gene signature
Down common gene signature
D
Fit score (a.u.)
+1.0
+0.5
0.0
−0.5
−1.0
**
**
Immature
TLX1
TLX3
HOXA
TAL-R
E
Enrichment plot:
“shared Vav1−/−/Zfp36l1−/−;Zfp36l2−/− gene signature” upregulated
NES: +2.02
FDR q-val: <0.001
TLX
(positively correlated)
Non-TLX
(negatively correlated)
Rank in Ordered Dataset
Enrichment profile
Hits
Ranking metric scores
Enrichment plot:
“shared Vav1−/−/Zfp36l1−/−;Zfp36l2−/− gene signature” downregulated
NES: −2.06
FDR q-val: <0.001
TLX
(positively correlated)
Non-TLX
(negatively correlated)
F
Upregulated genes
TLX+ tumor core enrichment
211
81
57
Immature T cell core enrichment
Downregulated genes
TLX+ tumor core enrichment
215
69
82
Immature T cell core enrichment
G
Dataset 2
VAV3
NOTCH2
HES1
MYC
CCNC
NOTCH3
PTCRA
NOTCH1
ZFP36L2
VAV2
VAV1
FBXW7
RUNX1
ZFP36L1
CBLB
+1.0
+0.5
0.0
−0.5
−1.0
Dataset 3
VAV3
RUNX1
ZFP36L1
ZFP36L2
MYC
CBLB
FBXW7
VAV1
NOTCH2
CCNC
NOTCH1
VAV2
NOTCH3
HES1
PTCRA

H

Figure	Protein	Lane	Mean value (a.u.)	p value	n
7H	Vav1	1	1.00±0.07	-	4
		2	0.15±0.04	0.0001 ***	4
		3	0.91±0.07	0.141	4
		4	1.08±0.02	0.097	4
		5	0.93±0.13	0.064	4
		6	0.32±0.09	0.0001 ***	4
		7	0.31±0.07	0.0001 ***	4
		8	0.24±0.17	0.0022 **	4
		9	1.00±0.09	-	3
		10	0.12±0.03	0.0001 ***	3
		11	0.29±0.11	0.0001 ***	3
		12	1.21±0.20	0.288	3
7I	Vav1	1	1.00±0.12	-	3
		2	1.09±0.09	0.255	3
		3	0.25±0.17	0.0001 ***	3
		4	1.22±0.07	0.0767	3
		5	0.15±0.04	0.0001 ***	3

Figure S7, related to Figure 7. The Vav1–ICN1 axis is downmodulated in human TLX^{+} T-ALL. **(A)** Scatter plot showing *VAV1* mRNA expression across indicated human T-ALL subtypes (bottom) and microarray datasets. Dots represent values from an individual patient sample. The mean and SEM for the overall sample set are also depicted. ***, p ≤ 0.001 (Tukey's HSD test). **(B)** Scatter plots showing *VAV1* mRNA abundance against the combined amount of *TLX1/TLX3* mRNA expression in indicated array datasets. Dots represent values from an individual sample. **(C)** ssGSEA-generated heatmap of the up- and downregulated "shared *Vav1*$^{-/-}$/*Zfp36l1*$^{-/-}$;*Zfp36l2*$^{-/-}$ gene signature" enrichment score for indicated T-ALL cases using microarray dataset 1. ssGSEA enrichment scores are depicted on a dark blue (lowest) to dark red (highest) scale. Samples with moderate and high signature fits are highlighted with grey and black bars, respectively. **(D)** Box plot of the "shared *Vav1*$^{-/-}$/*Zfp36l1*$^{-/-}$;*Zfp36l2*$^{-/-}$ gene signature" fit score for indicated T-ALL subtype samples (bottom) using microarray dataset 1. Data are represented as in **Figure 2C**. **, p ≤ 0.01 (Tukey's HSD test). **(E)** GSEA of the "shared *Vav1*$^{-/-}$/*Zfp36l1*$^{-/-}$;*Zfp36l2*$^{-/-}$ gene signature" in human T-ALL samples (TLX vs non-TLX tumors) using microarray dataset 1. The NES and FDR are indicated inside each GSEA graph. **(F)** Venn diagrams showing the overlap between the TLX-enriched and immature-T-cell enriched fractions of the "shared *Vav1*$^{-/-}$/*Zfp36l1*$^{-/-}$;*Zfp36l2*$^{-/-}$ gene signature". **(G)** Expression correlation matrix of TLX T-ALL samples positive for the "tumor-specific" *Vav1*$^{-/-}$/*Zfp36l1*$^{-/-}$;*Zfp36l2*$^{-/-}$ gene signature in indicated microarray datasets. Positive and negative correlation is shown in red and blue, respectively. The size of circles and color intensity are proportional to the Pearson correlation coefficient found for each transcript pair. Correlations with p values below the significance threshold of 0.05 (which relates with Pearson correlation coefficients above 0.33 and 0.28 in left and right panels, respectively) have been considered as statistically significant and labeled with asterisks. Genes encoding negative regulators of the Notch1 pathway and ICN1 targets are shown in red and blue letters, respectively. **(H)** Statistics of Western blot data shown in **Figure 7H** and **7I**. **, p ≤ 0.01; ***, p ≤ 0.001 (Student's t test).

A

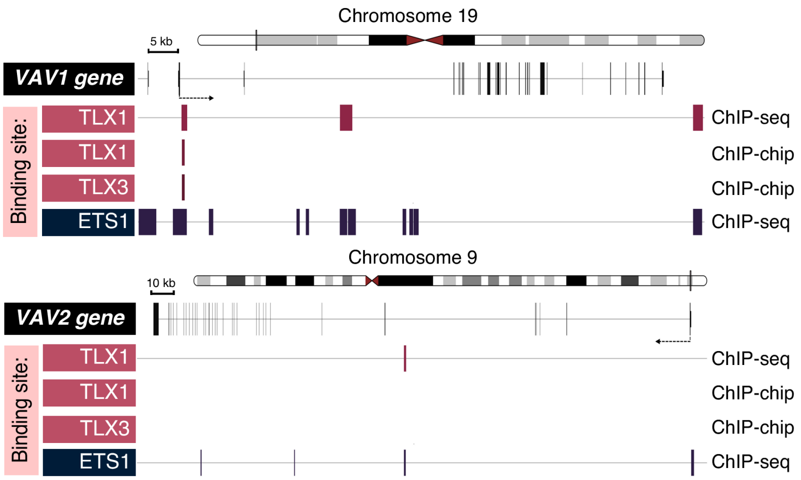

B

Figure	Protein	Lane	Mean value (a.u.)	p value	n
8A	Vav1	1	1.00±0.09	-	4
		2	0.33±0.11	0.007 **	4
		3	1.22±0.20	0.088	4
8A	ICN1	1	1.00±0.15	-	4
		2	2.66±0.37	0.0001 ***	4
		3	0.88±0.08	0.077	4
8A	Cbl-b	1	1.00±0.05	-	3
		2	1.09±0.07	0.211	3
		3	1.03±0.09	0.739	3
8B	Vav1	1	1.00±0.12	-	3
		2	2.49±0.44	0.0002 ***	3
8B	ICN1	1	1.00±0.23	-	3
		2	0.19±0.11	0.0001 ***	3
8B	Cbl-b	1	1.00±0.03	-	3
		2	0.89±0.13	0.098	3
8B	TLX1	1	1.00±0.10	-	3
		2	0.19±0.08	0.0001 ***	3
8C	Vav1	1	1.00±0.03	-	3
		2	3.11±0.28	0.0001 ***	3
8C	ICN1	1	1.00±0.08	-	3
		2	0.14±0.10	0.0001 ***	3
8C	Cbl-b	1	1.00±0.11	-	3
		2	0.91±0.12	0.188	3
8C	TLX3	1	1.00±0.07	-	3
		2	0.08±0.02	0.0001 ***	3
S8C	Vav1	1	1.00±0.08	-	3
		2	0.11±0.09	0.0001 ***	3
		3	0.12±0.11	0.0001 ***	3
		4	0.10±0.02	0.0001 ***	3
S8C	ICN1	1	1.00±0.16	-	3
		2	3.99±0.34	0.0001 ***	3
		3	3.23±0.55	0.0001 ***	3
		4	3.18±0.16	0.0001 ***	3

C

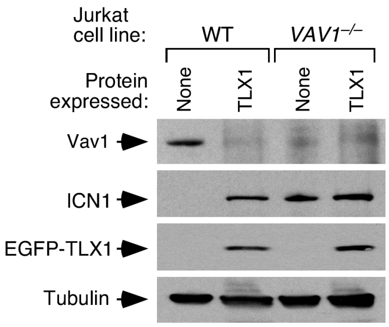

D

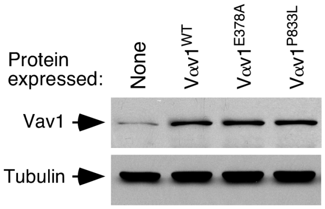

E

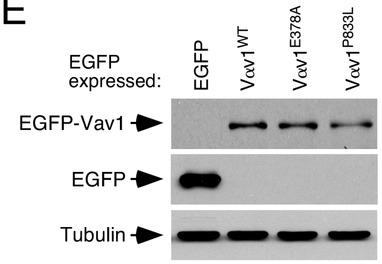

F

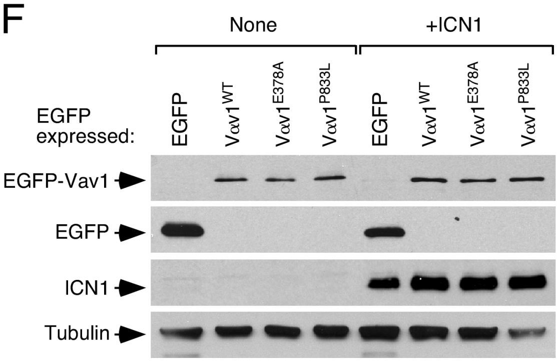

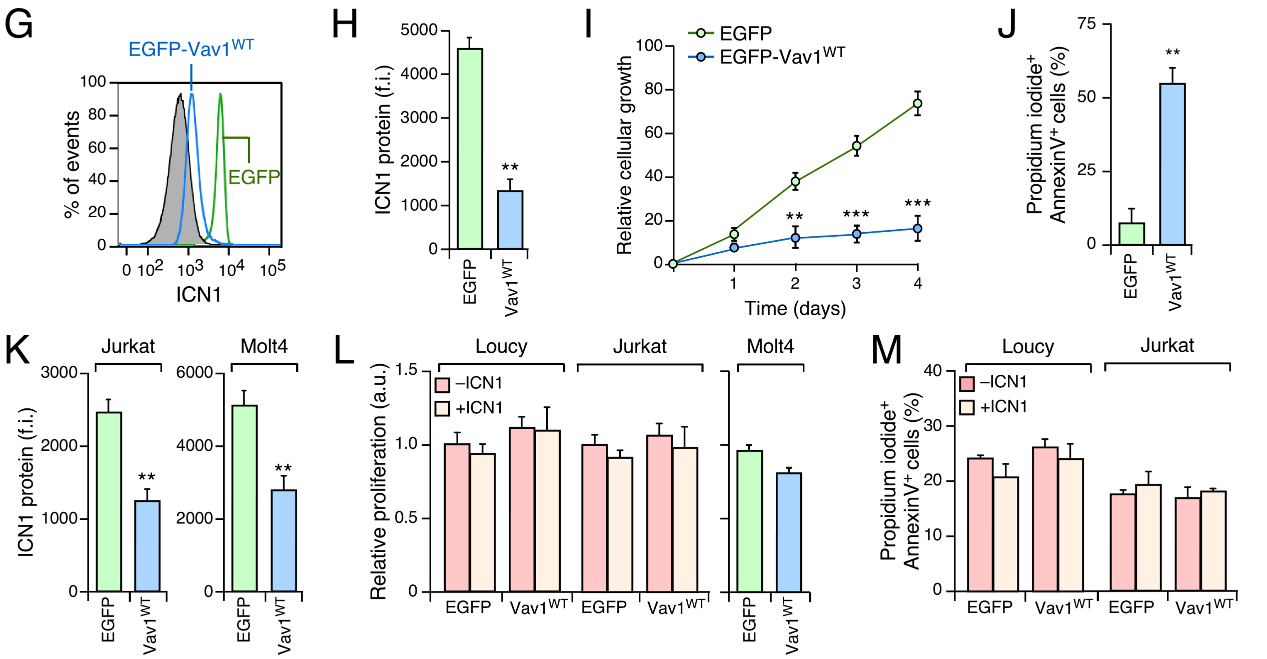

Figure S8, related to Figure 8. The TLX-mediated downmodulation of Vav1 is important for TLX$^+$ T-ALL pathogenesis. **(A)** Binding of indicated transcriptional factors (left) to regions present in *VAV1* and *VAV2*. Exons and transcriptional factor binding sites are shown in black and red boxes, respectively. Data about chromosomal localization (top), transcriptional origin (arrows), and locus scale (top) are also included. The type of experiment used to generate the data is shown on the right. **(B)** Statistics of Western blot data shown in indicated figures of this work. **(C)** Effect of the ectopic expression of EGFP-TLX1 in the abundance of endogenous Vav1 (top panel) and ICN1 (second panel from top) in TCLs from indicated Jurkat cells (top). Controls for the expression of the ectopic EGFPs (third panel from top, using antibodies to GFP) and endogenous tubulin α (bottom panel) are also shown. (**D-F**) Abundance of the indicated proteins (left) in TCLs obtained from the experiments shown in **Fig. 8F** (D), **8G** (E) and **8H-J** (F), respectively. In E and F, EGFPs were detected using antibodies to GFP. **(G-J)** Effect of the ectopic expression of indicated EGFPs in ICN1 abundance (G,H), growth (I), and apoptosis (J) of TLX1$^+$ ALL-SIL cells (n = 3). (**K-M**) Effect of the overexpression of EGFP and EGFP-Vav1^{WT} in endogenous ICN1 abundance (K), proliferation (L) and apoptosis (M) of indicated cells (top). The experiments were carried out either the absence (–ICN1) or presence (+ICN1) of ectopically expressed ICN1 (n = 3). In panels B and H to M, data represent the mean ± SEM. **, p ≤ 0.01; ***, p ≤ 0.001 (Student's t tests).

Table S4, related to Star Methods. List of primers used for the generation of indicated mutant proteins

Gene	Primer	Primer sequence
Mouse *Vav1* (NM_011691)	E378A_Fw	5'-GAGGTCAAGAGGGACAATGCAACCCTACGGCAGATCACA-3'
	E378A_Rv	5'-TGTGATCTGCCGTAGGGTTGCATTGTCCCTCTTGACCTC-3'
	L334A_Fw	5'-CCTATGCAGCGGGTGGCGAAGTACCACCTCCTT-3'
	L334A_Rv	5'-AAGGAGGTGGTACTTCGCCACCCGCTGCATAGG-3'
	R375A_Fw	5'-GTGAACGAGGTCAAGGCCGACAATGAAACCTGA-3'
	R375A_Rv	5'-TAGGGTTTCATTGTCCGCCTTGACCTCGTTCAC-3'
Human *CBLB* (U26710)	Y363F_Fw	5'-CAGGAACAATATGAATTATTTTGTGAAATGGGCTCCACT-3'
	Y363F_Rv	5'-TCACCTCGGGTAAAGTGTTTTATTAAGTATAACAAGGAC-3'
	PRRmut1_Fw	5'-GGTGAGAAAACAAGATAAAGCCACTCCCAGCACCACCTC-3'
	PRRmut1_Rv	5'-CTCCACCACGACCCTCACCGAAATAGAACAAAAGAGTGG-3'
	PRRmut2_Fw	5'-GAGATCCTCCTCCACCGCCACTGAAAGACCTCCACCAATC-3'
	PRRmut2_Rv	5'-CTAACCACCTCCAGAAAGTCACCGCCACCTCCTCCTAGAG-3'
Mouse *Notch1* (NM_008714)	ΔANK7_Fw	5'-CAACCAGACAGACCGCACCTGAGAGACCGCCTTGCACTTG-3'
	ΔANK7_Rv	5'-GTTCACGTTCCGCCAGAGAGTCCACGCCAGACAGACCAAC-3'
	ΔANK6_Fw	5'-CTGAAGAACGGAGCCAACAAGGACATCGAGAACAACAAG-3'
	ΔANK6_Rv	5'-GAACAACAAGAGCTACAGGAACAACCGAGGCAAGAAGTC-3'
	ΔANK5_Fw	5'-GCCGTGGATGACCTAGGCTAGTCGGCTTTGCATTGGGCG-3'
	ΔANK5_Rv	5'-GCGGGTTACGTTTCGGCTGATCGGATCCAGTAGGTGCCG-3'
	ΔANK4_Fw	5'-CTGAAGAACGGAGCCAACTAGGACATCGAGAACAACAAG-3'
	ΔANK4_Rv	5'-GAACAACAAGAGCTACAGGATCAACCGAGGCAAGAAGTC-3'
	ΔANK2_Fw	5'-CAACCAGACAGACCGCACCTGAGAGACCGCCTTGCACTTG-3'
	ΔANK2_Rv	5'-GTTCACGTTCCGCCAGAGAGTCCACGCCAGACAGACCAAC-3'
	A2060V_Fw	5'-GCTTTGCATTGGGCGGCCGTGGTGAACAATGTGGATGCTG-3'
	A2060V_Rv	5'-GTCGTAGGTGTAACAAGTGGTGCCGGCGGGTTACGTTTCG-3'
